# Fragment-based Shape Signatures: a new tool for virtual screening and drug discovery

**DOI:** 10.1007/s10822-013-9698-7

**Published:** 2013-12-24

**Authors:** Randy J. Zauhar, Eleonora Gianti, William J. Welsh

**Affiliations:** 1 Department of Chemistry and Biochemistry, University of the Sciences, 600 S. 43rd Street, Philadelphia, PA 19104 USA; 2 Department of Pharmacology, Robert Wood Johnson Medical School, University of Medicine and Dentistry of New Jersey, 675 Hoes Lane, Piscataway, NJ 08854 USA

**Keywords:** Ligand-based drug design, Fragment-based shape signatures, Scaffold hopping, Shape signatures, Shape similarity, Virtual screening

## Abstract

Since its introduction in 2003, the Shape Signatures method has been successfully applied in a number of drug design projects. Because it uses a ray-tracing approach to directly measure molecular shape and properties (as opposed to relying on chemical structure), it excels at scaffold hopping, and is extraordinarily easy to use. Despite its advantages, a significant drawback of the method has hampered its application to certain classes of problems; namely, when the chemical structures considered are large and contain heterogeneous ring-systems, the method produces descriptors that tend to merely measure the overall size of the molecule, and begin to lose selective power. To remedy this, the approach has been reformulated to automatically decompose compounds into fragments using ring systems as anchors, and to likewise partition the ray-trace in accordance with the fragment assignments. Subsequently, descriptors are generated that are fragment-based, and query and target molecules are compared by mapping query fragments onto target fragments in all ways consistent with the underlying chemical connectivity. This has proven to greatly extend the selective power of the method, while maintaining the ease of use and scaffold-hopping capabilities that characterized the original implementation. In this work, we provide a full conceptual description of the next generation Shape Signatures, and we underline the advantages of the method by discussing its practical applications to ligand-based virtual screening. The new approach can also be applied in receptor-based mode, where protein-binding sites (partitioned into subsites) can be matched against the new fragment-based Shape Signatures descriptors of library compounds.

**Electronic supplementary material:**

The online version of this article (doi:10.1007/s10822-013-9698-7) contains supplementary material, which is available to authorized users.

## Introduction

Molecular shape remains the fundamental determinant in our understanding of the mechanisms of bioactivity [1]. The specificity of interactions between ligand and receptor is largely defined by shape and electrostatic complementarity, with shape the most critical factor (in that a small addition of steric bulk can dramatically increase interaction energy). Both ligand- and receptor-based approaches to computer-aided drug design take shape into account either implicitly, by assuming that similar chemical structures will have similar interactions with a target (as typified by pharmacophore screening methods [2, 3]), or explicitly by fitting ligands into the volume presented by a binding site (as seen in molecular docking [4–6]). In either case, shape is a preeminent consideration, and given the rapid increase in the size of available chemical libraries, it remains a challenge to efficiently screen large compound databases to identify compounds likely to evince shape similar to a query molecule, or to match the complementary volume of a protein receptor.

Another argument that draws increasing attention is the practical issue of securing intellectual property rights for potential therapeutics. Once a concern primarily in industry, this has now become a pressing consideration for academic scientists as universities seek new sources of funding, and see attractive possibilities in commercializing basic research [7]. This increases the value of computational strategies that support “scaffold hopping”, the ability to rapidly expand the chemical space being explored. To accomplish this, a conventional ligand-based search strategy that relies on structure-based descriptors must either cast a very wide net (e.g. using physicochemical parameters as filters), or consider at the outset a broad range of alternative structural classes that will likely be suggested by chemical intuition and/or synthetic feasibility. In the first case, most molecules collected will be false positives, and their number will exceed the capacity of typical secondary screens (such as molecular docking), while in the latter case the chemical space considered will inevitably be restricted by the initial choices made, reducing the chances of identifying truly novel compounds. Methods that rely on the development of structural queries also presume a high level of chemical expertise, and may be difficult for the non-computational specialist to apply.

Since its introduction in 2003, the Shape Signatures method [8, 9] has proven a useful tool in a number of drug discovery projects [10–12] (including several proprietary investigations). The Shape Signatures technique uses a simple implementation of *ray*-*tracing* [13], a method borrowed from computer graphics imaging, to stochastically explore the volume enclosed by the solvent-accessible surface (SAS) of a ligand molecule, or the volume exterior to a protein receptor site. Once generated, probability distributions are derived from the ray-trace and stored as histograms; these are the Shape Signatures. While the ray-tracing operation is computationally challenging, it need be carried out only once for each library compound, and the Shape Signatures descriptors are then rapidly compared, with speed comparable to chemical fingerprint methods. Moreover, a number of descriptors are generated from a single ray-trace, which are classified as “1D” or “2D” according to the dimension of the domain of the associated probability distribution (histogram). The single 1D descriptor generated in the current implementation is simply the distribution of ray-trace segment lengths, while the 2D descriptors represent joint probability distributions that couple shape with electrostatic potential information sampled on the molecular surface (described in “Methods”).

Shape Signatures present a number of attractive advantages over other methods. First, it depends explicitly on shape, not on the underlying chemical structure, and thus excels at scaffold hopping; moreover, the Shape Signatures descriptors have been proven to be very sensitive to the details of molecular shape, while less so on conformation, reducing the need for preprocessing of query structures (e.g., in general, multiple conformers do not need to be generated for a query molecule). Secondly, the method is fast, with performance comparable to chemical fingerprints, and offers the capability to scan a library comprising millions of compounds in a matter of minutes. Thirdly, the method unifies ligand- and receptor-based approaches, since one has the option of comparing the shapes of molecules against other molecules (shape similarity), or molecules against a receptor site (shape complementarity). Finally, running searches is remarkably easy, requiring only that the end user supply a query structure and runtime parameters to control the number of hits returned.

**Fig. 1:**
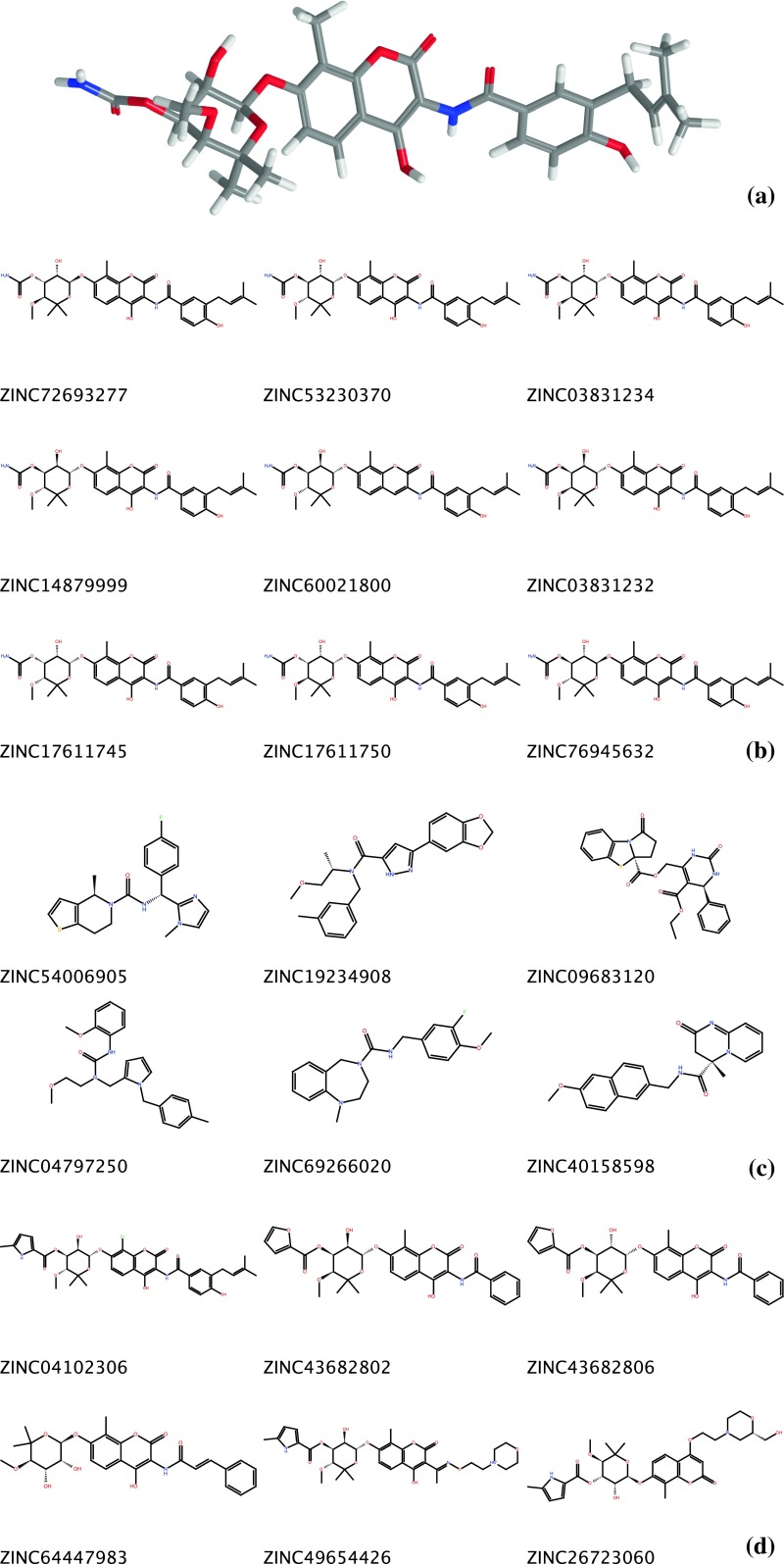
**a** Extended conformation of Novobiocin. A Shape Signature descriptor was prepared from this structure and used as query against the ZINC database. **b** Top hits for Novobiocin. All of these are structurally identical to the query, although reflecting minor changes in conformation (e.g. changes in conformation of hexose ring with respect to the query). **c** Novobiocin hits 21–26. These are roughly similar in size to the query, but lack significant shape matches involving ring systems or substituents. **d** Compounds ranked much lower in the hit the list, which do exhibit interesting substructural similarity to the query. These molecules all feature a hexose moiety and a coumarin derivative, while including terminal substituents or linkers that deviate from the query. The rank positions range from 183 (ZINC43682806) to 6,118 (ZINC26723060)

Despite these advantages, Shape Signatures has suffered from an important drawback—as one moves from query compounds based on one or two ring systems to more complicated and heterogeneous molecules, the selective power of the method degrades. This is perhaps an unavoidable side-effect of the original implementation of the method, where all of the shape information for a molecule is compressed into a very compact descriptor. To illustrate, we scan the ZINC [14, 15] library with an extended conformer of the antibiotic Novobiocin, which comprises rings of three distinct classes (phenol, coumarin and hexose) along with diverse substituents (Fig. 1a). The query molecule itself (present in ZINC in multiple copies, along with close structural analogs) does appear at the top of the hit list (Fig. 1b), but moving down past the top ten molecules we encounter hits that bear little resemblance to the query, neither in the ring systems they include nor in overall topology (Fig. 1c). While exploration of chemical diversity is an important feature of the Shape Signatures approach, hits that match the query only in overall size can be more easily identified by simple property queries. While interesting hits that feature significant similarity to the original query do appear in the hit list (Fig. 1d), many have poor rank. Shape Signatures remains a useful tool (in conjunction with other screens) when applied to complex molecules like Novobiocin, but it becomes necessary to retain a very large hit list, and the advantages of the method over competing techniques become less apparent.

To remedy this deficiency, we have extensively modified the original algorithm so that it is now *fragment*-*based*. As described below, we retain the ray-tracing approach of the original method, but now automatically partition molecules into fragments based on ring systems (or by a custom specification provided by the user). The ray-trace is likewise partitioned in accordance with the fragments, and the Shape Signatures descriptors are expressed as an array of inter- and intra-fragment contributions. When a query molecule is compared to a database target compound, the query fragments are mapped onto the target fragments in all ways consistent with the underlying connectivity (including substructure matches). Fragment-based comparison scores are generated, which involve a weighted average of the fragment–fragment contributions defined by the mapping, and which are used to rank hits in order of significance. In this way, the selective power of the method (which holds only when well-defined shapes are represented) is carried over to larger and more complex structures, with the added bonus that query and target structures are mapped onto each other in chemically meaningful ways. We stress that while mappings must be consistent with underlying chemical connectivity, the fragment-based Shape Signatures descriptors are still based only on fragment shape, not underlying chemical structure, so we expect the scaffold-hopping capabilities of the method to be largely preserved.

In what follows we will describe in detail our new implementation. We will discuss typical applications of the new approach, and present a basic validation study.

## Methods

### Automatic fragmentation

**Fig. 2:**
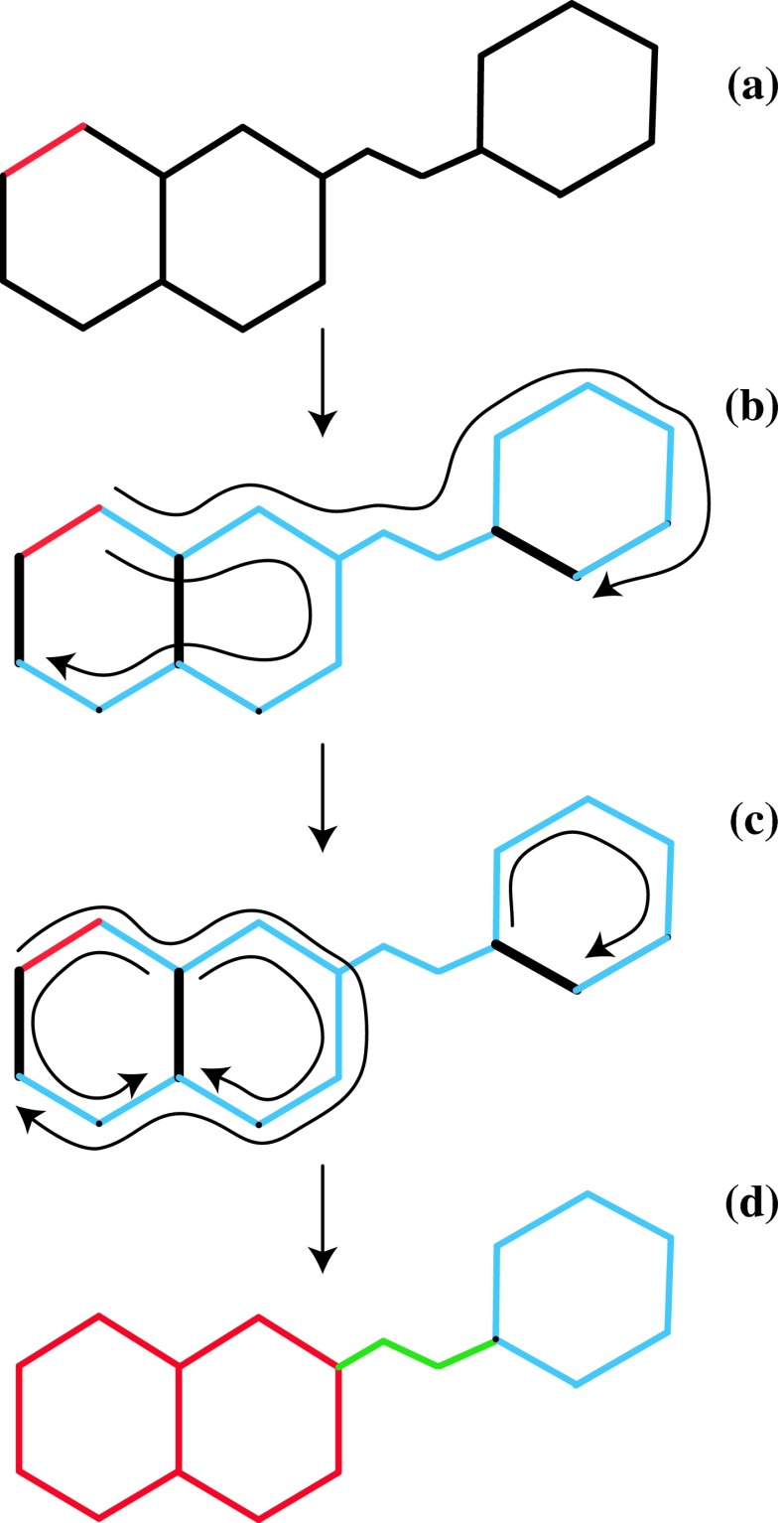
**a** Input structure. One bond (highlighted in *red*) is selected at random as the initial seed. **b** Paths (*curved black*) are launched from one end of the seed bond and propagated recursively. A path is terminated if continuation would mean contacting an existing portion of the same path. The terminating bonds discovered by the paths are identified as ring closures (*heavy black*). **c** Paths are launched recursively from the ends of the ring closures; unions of non-self-intersecting paths that connect one end of the ring closure to the other identify ring-based fragments. **d** After ring based fragments (*colored red* and *blue*) are defined, remaining connected components of the molecule define terminal or internal non-ring fragments (*green*)

The basis of our new approach is to fragment molecules automatically using a simple and robust algorithm (the method is illustrated in Fig. 2). The key idea (which is certainly not novel) is to break molecules up based on ring systems. The first step is to identify ring closures, which are equal in number to the cycles in the graph of the molecule (constructed by treating heavy atoms as vertices and bonds as edges). Closures are located by choosing any bond as an initial seed for a subtree, and extending the subtree recursively to include neighboring bonded vertices, but excluding neighbors that have already been incorporated into the tree. When the recursion terminates the subtree is *maximal* [16], and any remaining bonds not incorporated into the tree are ring closures (Fig. 2a). Ring closures are guaranteed to be equal in number to the cycles formed by the graph, but their positions are not uniquely determined and depend upon the specific path of execution taken by the algorithm.

Next, paths of bonds are generated recursively, using ring closures as initiation points, and with paths extended using all contiguous non-closure bonds. The union of all paths that end at the closure initiation point define a single ring system (Fig. 2b). As shown in the diagram, a fused ring system will include multiple ring closures, and these are eliminated from further consideration once incorporated into the union of paths associated with any other closure in the same ring system. Once all ring closures have been processed in this way, the molecule is partitioned into an initial collection of identified ring systems, along with the remaining non-ring components (at this stage only heavy atoms have been considered).

Connected components of the heavy-atom graph are next identified, and these belong to one of the following categories: (a) a ring system, (b) a fragment neighboring two or more ring systems, or (c) a fragment neighboring a single ring system. Components that border two or more ring systems are always assigned as separate fragments; components that border a single ring system are assigned as separate fragments if they contain more than five heavy atoms, and are subsumed into the neighboring ring system if smaller. Finally, hydrogens are assigned to the fragments they are attached to.

As an alternative to the automatic procedure just described, it is also possible for the user to supply a file that assigns atoms (by index) to numbered fragments. This is useful in cases where some criteria other than ring system membership is preferred for defining fragments (e.g. when considering peptides or other polymers), and is a prerequisite if the fragment-based approach is to be applied to exterior ray-traces for receptors. In receptor-based modeling, “fragments” correspond to sub-sites of the binding site, and we assume these must be defined by the end user. While the symmetric handling of ligands and receptors is an attractive feature of Shape Signatures, here we will discuss only the ligand-based application of the method.

### Surface generation

**Fig. 3:**
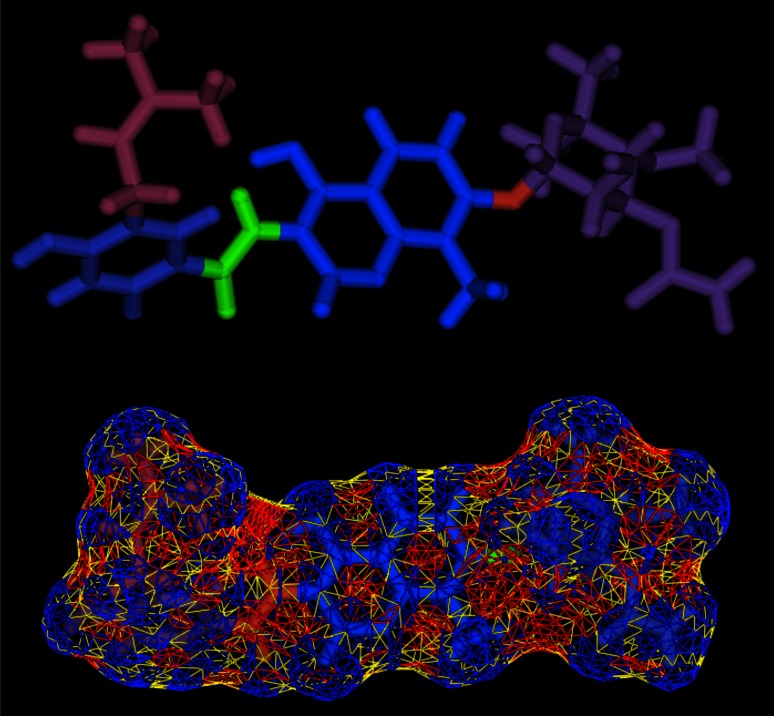
Triangulated surface for Novobiocin generated by Smooth Molecular Surface Triangulator (SMART). *Top*: Starting structure, with fragment assignment indicated by color coding. *Bottom*: Triangulated surface. The surface elements are *color*-*coded* using the Connolly/Richards convention of assigning contact (*blue*), saddle (*yellow*) and reentrant (*red*) surface categories

The shape of a molecule in Shape Signatures is described by its SAS, which is generated using the inward face of a rolling probe (representing a single solvent molecule), as originally described by Lee and Richards [17] and later implemented in widely-used algorithms by Connolly [18]. In this work, an updated version of the SMART algorithm [19] is applied, which generates a triangulated surface that closely matches the ideal mathematical form of the SAS. Although the updated algorithm includes a new approach to dealing with potentially self-intersecting surface, for the purposes of Shape Signatures this modification is unimportant and should not significantly influence results. Figure 3 shows a triangulated surface for Novobiocin generated using the updated SMART tool. Atomic coordinates for SMART are taken directly from database entries if available; if the structure format does not define explicit coordinates, these are first generated using CORINA (http://www.molecular-networks.com).

### Ray-tracing

The ray-tracing algorithm is similar to that of the original Shape Signatures code [8], but has been re-implemented and updated to take into account partitioning of molecules into fragments. Briefly, a rectangular grid of specified spacing is first overlaid on the triangulated surface of the molecule, and surface elements are assigned to cubes of this acceleration grid [20] based on contact with the element corners—if any corner of an element lies within the boundaries of a grid cube, then the element is assigned to that cube. Thus an element can be assigned to a minimum of one cube, but to no more than three. Surface elements are also assigned to atoms, and the number of assigned atoms depends on the class of molecular surface (contact, saddle of reentrant) the element belongs to [18]. (Briefly, elements of contact surface are assigned to one atom, saddle elements to two, and reentrant elements to three atoms.) Moreover, each element is assigned to a single principal atom, namely the one closest to the geometric center of the element.

The ray-trace is initiated at a randomly chosen element that forms part of the contact surface of the molecule. Contact surface is found where the solvent probe slides with two degrees of freedom (locating a fully solvent-exposed portion of an atom), and this class of surface is unlikely to involve any feature (e.g. a narrow invagination) that might inhibit the initial propagation of the ray. The ray is started at the center of the element, and perpendicular to the element plane. For ligand-based applications the goal is to explore the geometry defined by the interior of the surface, and the ray is initially propagated antiparallel to the outward-directed surface normal vector. On the other hand, when characterizing the shape of a protein receptor site the ray is initially directed in the same direction as the surface normal vector, so the ray-trace inhabits the volume exterior to the molecular surface. When performing a receptor-based ray-trace, the initial element is chosen at random from contact elements belonging to atoms of the binding site, which is defined by the user.

**Fig. 4:**
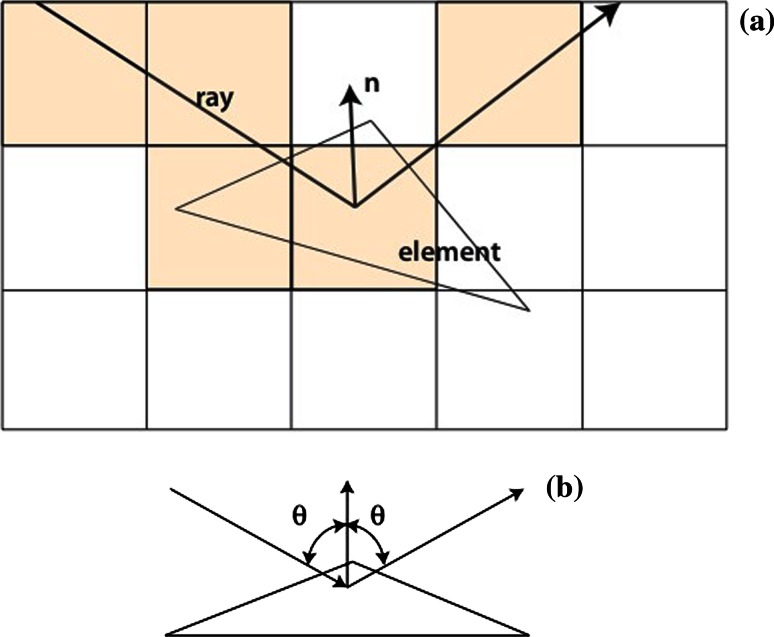
Geometry of ray-tracing. **a** Cubes of the acceleration grid that the propagating ray passes through are shaded in this 2D illustration. Each cube may be associated with surface elements, and the elements attached to any cubes touched by the ray are checked for possible intersection with the ray. The first intersection detected along the path of a ray locates the next reflection. **b** The direction of the reflected ray is defined by the vector normal to the surface (**n**), with the normal, incident and reflecting rays all lying in the same plane, and with equal angles between the normal vector and the incoming and reflected rays

Once started, the ray propagates by the rules of optical reflection. (The polygonal triangulated molecule can be imagined as a sort of distorted mirrored disco ball for the purpose of visualization.) Progress of the ray is tracked through the cubes of the acceleration grid, and each cube encountered is checked for associated surface elements; if the cube is nonempty, each surface element it contains is tested to see if it supports a reflection, and the computed reflection points are ordered by position along the ray. The reflection point closest to the previous reflection point (or the initiation point, if at the start of the trace) terminates the current ray-trace *segment*, and defines the start of the next segment (Fig. 4). The direction of the new segment is determined by the angle of incidence at the reflecting element, plus a random perturbation that is uniformly distributed within a cone centered on the ray direction that corresponds to ideal reflection. The angle of the cone is a user-supplied parameter (typically chosen as 5° or less), and this random perturbation serves to prevent the ray from ever becoming “stuck” for a long period between opposing surface elements with antiparallel normals.

If an exterior ray-trace is being performed for a protein receptor site, the ray-trace is restricted to those atoms in the user-defined site (which is specified by supplying a text file containing the site-atom indices). This is accomplished by simply truncating the trace if it encounters a non-site surface element, and reinitiating at a randomly-chosen position within the site; the ray-trace segments leading up to the escape of the ray are retained. Similarly, in the rare case that normal propagation of the ray-trace fails (sometimes observed if a ray exactly intersects the boundary between two elements), then the ray-trace is simply truncated at the last successful reflection, and the algorithm is restarted.

In addition to the position of each reflection, we also retain the molecular electrostatic potential (MEP) (which is interpolated from the values pre-computed at the vertices of the incident surface element, using imported partial atomic charges). MEP values are used in the construction of “2D” Shape Signatures, as described in the next section.

Each segment of the ray-trace is associated with up to two atoms, and we presume these have been assigned to numbered molecular fragments. A ray-trace segment is categorized as intra- or inter-fragment depending upon whether the two atoms belong to the same or different fragments, and the fragment identities of each segment are retained for later analysis.

### Shape Signatures descriptors

**Fig. 5:**
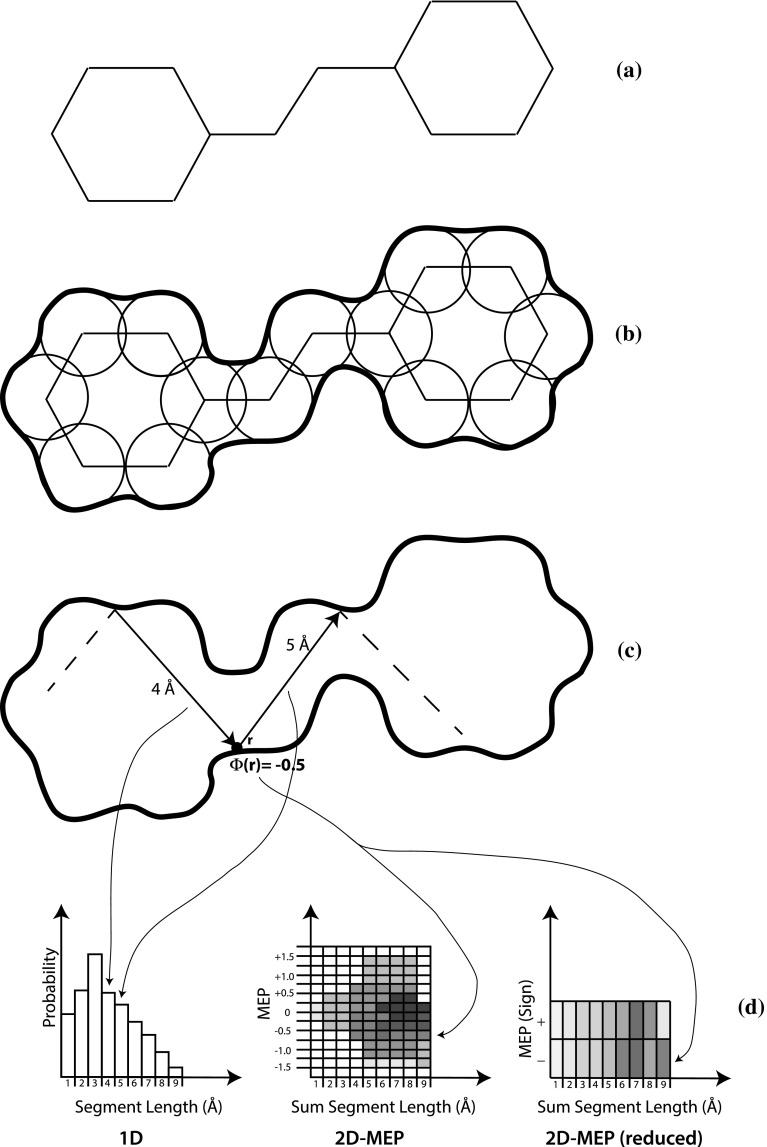
Computational flow to generate Shape Signatures descriptors. **a** Input molecular structure. **b** Assignment of atomic radii, generation of solvent-accessible surface using rolling probe (*heavy outline*). **c** Ray trace; two successive segments are show, involving a reflection at position **r**. The incoming segment has length 4 Å, the segment leaving the reflection has length 5 Å. The molecular electrostatic potential (MEP) at position **r** (computed from atomic partial charges) is −0.5 **d** Shape Signatures descriptors. The “1D” descriptor accumulates the distribution of ray-trace segment lengths, approximated by a histogram. The “2D” descriptors accumulate the joint probability of observing a sum of segment lengths centered at a reflection, coupled with the MEP measured at the reflection point. For the 2D-MEP descriptor the numerical value of the MEP is used directly to identify a histogram bin, for the “reduced” descriptor only the sign of the potential is taken into account

The Shape Signatures descriptors are histograms that accumulate selected probabilities derived from the ray-trace. “1D” signatures correspond to probability distribution with one-dimensional domain, while “2D” descriptors are joint probability distributions with two-dimensional domain. (Signatures with higher dimension are possible, but have not been actively investigated yet.) Figure 5 summarizes the signatures currently implemented.

The simplest 1D descriptor is the distribution of ray-trace segment lengths, which is easily computed from the ray-trace reflection positions given a histogram bin width; the bin-width parameter is user-supplied, but must match the value used to construct target databases, and is thus not freely selectable. The first 2D signature currently supported corresponds to the joint probability distribution that describes the sum of the segment lengths incident at a given reflection point, together with the MEP computed at the reflection point (Fig. 5d). Two variants of this basic signature type are also computed: a *reduced* 2D descriptor, which includes only two bins for the electrostatic potential, corresponding to positive or negative sign, and a *reduced inverted* descriptor (not shown), again with two electrostatic bins but with the sign of the potential reversed. The motivation for the reduced descriptors is our recognition that it is overly restrictive to require identical MEP distributions over the surfaces of two molecules in order to deem them similar; having the same sign of the potential is adequate to suggest a useful match. The inverted 2D descriptor is to support receptor-based applications, where one is interested in identifying small molecules that are complementary in shape *and* electrostatic potential to a target; by generating a descriptor with the sign of the potential inverted “up front”, it is easier to use the same scoring machinery (described below) to evaluate complementary electrostatic matches.

**Fig. 6:**
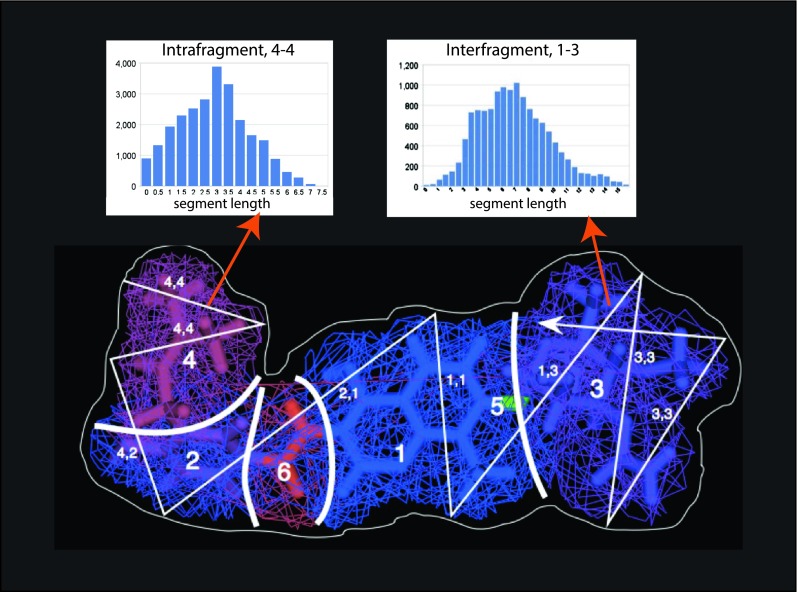
Generation of fragmented Shape Signature descriptor histograms. Here ray-trace segments are color-coded using the same scheme as the molecular fragments. A segment whose end-points lie in the same fragment contributes to the appropriate intra-fragment histogram, while a segment with reflections in two distinct fragments contributes to the corresponding inter-fragment histogram

In accumulating the descriptor probability distributions, we take into account the fragment identification of the ray-trace segments, and maintain separate histograms for intra- and inter-fragment contributions (illustrated in Fig. 6). This is readily achieved, as each reflection is supported by a surface element, which in turn is associated with a primary atom, which is in turn a member of a fragment. Ray-trace segments with reflections in the same fragment contribute to an intra-fragment histogram; segments with reflection points in distinct fragments contribute to an inter-fragment histogram. The original, “global” histogram for all segments can be recovered by summing corresponding bins for all the intra- and inter-fragment histograms. Using the global histogram when scoring reproduces the original Shape Signatures approach, while the intra-fragment histograms serve as descriptors for the individual fragments that comprise the molecule. While the inter-fragment histograms represent a significant fraction of the ray-trace segments (typically about 20 %), and undoubtedly capture important aspects of molecular shape, we do not consider them at present when comparing molecules. They are nonetheless retained along with the intra-fragment descriptors and are available for use in improved scoring methods yet to be developed.

### Scoring

We retain the basic scoring approach of the original Shape Signatures method, and apply a discretized version of the conventional *L*_1_ metric to compute the distance between two histograms:

1 $$S(q,t) = \sum\limits_{\mu } {\left| {h_{\mu }^{q} - h_{\mu }^{t} } \right|}$$

here $h_{\mu }^{q}$ is the height of query histogram *q* at bin μ, and it is assumed that μ varies over corresponding bins (height, and also MEP in the case of 2D histograms). If the histograms are normalized (the case here), then *S(q,t)* is expressed in units of probability. The minimum score possible is zero, corresponding to identical histograms, and the maximum is 2, the case for two distributions with no common support.

**Fig. 7:**
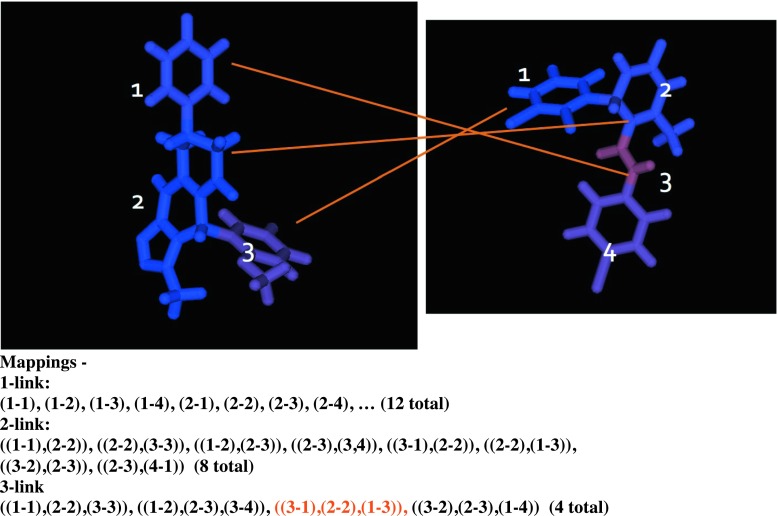
Possible mappings between a query with three fragments and a target compound with four. The text corresponding to the illustrated mapping is highlighted. Since our fragment mapping algorithm is greedy, not all of the mappings suggested here would be retained; for example, the “seed” mapping (1–2) would be expanded to ((1–2),(2–3))

We now extend this approach to handle fragment-based scoring. We assume a mapping *M(q,t)* that maps the set of fragments $\left\{ { \, f_{j}^{{^{q} }} } \right\}$ in the query molecule to fragments $\left\{ {f_{\alpha }^{t} } \right\}$ in the target (where the subscripts range over the available fragment indices in each molecule). Mappings must be consistent with the underlying chemical connectivity, in the sense that if query molecule fragments *x* and *y* are connected by a bond, and $f_{x}^{q}$ maps to $f_{\mu }^{t}$ while $f_{y}^{q}$ maps to $f_{\nu }^{t}$, then this implies that fragments μ and ν are bonded in the target molecule. Also, it is not required that all fragments in query and target be covered by the mapping (i.e. substructure matches are supported). The mapping algorithm we employ starts with the set of all possible mappings between single fragments in query and target, and recursively expands these in all ways consistent with fragment connectivity, and with duplicates detected and removed. The mapping expansion is “greedy” in that mappings are retained only after maximal extension (e.g. the initial single mappings between individual fragments are not retained). Figure 7 illustrates the possible mappings between two small molecules comprising multiple fragments.

Fragment-based scoring involves a straightforward extension of Eq. 1

2 $$S(q,t/M) = \frac{1}{{C^{q} + C^{t} }}\sum\limits_{m \in M} {\left( {C_{x(m)}^{q} + C_{\mu (m)}^{t} } \right) \cdot S\left( {f_{x(m)}^{q} ,f_{\mu (m)}^{t} } \right)}$$

where

3 $$C^{q} = \sum\limits_{m \in M} {C_{x(m)}^{q} } ,C^{t} = \sum\limits_{m \in M} {C_{\mu (m)}^{t} }$$

In Eq. 2, the sum is over all fragment–fragment links *m* in mapping *M,* from query molecule *q* to target *t*. Each query fragment $f_{x(m)}^{q}$ of mapping element *m* contains $c_{x(m)}^{q}$ intra-fragment ray-trace segments, similarly there are $c_{\mu (m)}^{t}$ segments associated with corresponding target fragment $f_{\mu (m)}^{t}$. The existing scoring function *S()* is applied to each pair of mapped fragments, and the resulting total score is simply the sum of these contributions, weighted by the number of ray-trace segments associated with the fragments being compared.

A single comparison between two molecules will in general produce a number of mappings, the specific number determined by both the number of fragments in query and target molecules, and their topology. The “hits” are ranked in order of ascending score (again, low scores indicate greater similarity), and a hit record also retains the details of the mapping between query and target, and the percentages of ray-trace segments *unused* in query and target. The latter numbers correlate with the volumes of the fragments excluded when forming the match, and these can be used in downstream filtering to remove substructure matches that involve too few atoms to be interesting.

### Generating descriptors for the ZINC database

Shape Signatures comparisons are rapid (even though fragment-based scoring is inevitably slower than the original approach). In contrast the ray-tracing operation is time-consuming even with grid acceleration in place, requiring on the order of 1 s CPU time for each molecule. That said, the computation of Shape Signatures is a one-time investment for a given target database, suggesting the most efficient approach is to gain short-term access to a massive computing resource to carry out descriptor generation for the database, after which more modest computing facilities will suffice to carry out searches.

In recent years, “cloud computing” has been recognized as an attractive solution in those situations where computational requirements can change dramatically in a short time frame [21] (e.g. in electronic commerce applications where customer demand can fluctuate dramatically as a function of the season or even time of day). In a cloud computing environment, data centers provide access to virtual machines that are distributed across the hardware in their facilities, and which can run a variety of operating systems; in addition, there will typically be available a distributed storage system which uses data redundancy to reduce access time and ensure reliability and fault tolerance. Perhaps the most successful and widely-used cloud computing environment is Amazon Web Services (AWS) which includes both a distributed storage service (“Simple Storage Service”, or S3) and an environment for configuring and deploying virtual machines (“Elastic Compute Cloud”, or EC2).

We have used AWS to generate Shape Signatures descriptors for version 11 of the ZINC database [14, 15]. ZINC is a library that comprises the major public resources (PubChem [22], NCI [23]) along with the catalogs of the major chemical vendors (e.g. SigmaAldrich (http://www.sigmaaldrich.com), Maybridge (http://www.maybridge.com)) and smaller specialty suppliers. ZINC covers a very large chemical space, includes many drug-like molecules, and generally features compounds likely to be readily acquired; thus it is an ideal resource for identifying compounds as early leads against a biological target of interest. While intellectual property rights would be limited if a ZINC compound were introduced unmodified as a therapeutic agent, active compounds identified in ZINC can serve as a testing ground for structure–activity hypotheses, and provide the starting point for synthetic strategies aimed at developing truly novel molecules.

Our calculations were carried out using custom Python scripts along with Boto (https://github.com/boto/boto, http://boto.readthedocs.org) an object-oriented Python toolkit which facilitates easy access to the S3 and EC2 features of AWS. We began by manually configuring a single Linux machine instance with requisite libraries, scripts and command-line tools. This machine instance was archived, and we could then deploy as many instances of the machine as needed. The ZINC database was downloaded as individual component libraries, and each of these in turn divided into multiple multi-mol2 files (http://www.tripos.com/data/support/mol2.pdf), each containing about 20,000 molecules. To reduce cost, we allocated machines using “spot requests”, where a price per hour of CPU activity is offered by the user (and must be accepted by AWS) before each machine is started. While this generally ensures a cost-effective usage rate, there is the risk that the machine will be prematurely terminated if demand rises and the adjusted minimum price exceeds the initially-accepted offer. To mitigate the possible loss of calculations, we subdivided each multi-mol2 file into 10 smaller units of about 2,000 molecules, and furthermore grouped each collection of 10 units into five pairs; a pair (comprising ~4,000 molecules) was submitted to a single two-core virtual machine for processing, and this required about 3–4 h of wall-clock time. For the initial calculations a spot-request price of $0.10/machine-hr was always adequate, but with increasing demand the threshold had to be raised to $0.15/machine-hr for calculations to run. There were occasional lapses in service, and a number of calculations failed due to timeouts when attempting to access input data, which was stored in S3. It was critical to carefully review the output for missing results, and to reschedule failed computations.

The output descriptors were computed using a custom command-line program written in objective-C, an object-oriented extension of the C language (developer.apple.com). The Linux implementation relies heavily on the GNU port of the NextStep Foundation classes (http://www.gnustep.org) to support complex data structures and for access to machinery for archiving, and for access to operating system resources. The Shape Signature descriptors were saved in a custom compressed XML format, and the results generated by each virtual machine were archived, compressed and transferred for storage to S3.

The Shape Signatures descriptors generated on EC2 virtual machines and stored in S3 were finally transferred to a single server (artemisdiscovery.com) and imported as binary objects into a MySQL (www.mysql.com) database. The use of a relational database allows for rapid random-access retrieval of signatures, enables easy duplicate checking (critical for us, since the compound memberships of the component ZINC libraries significantly intersect), and provides an easy route to attaching new descriptor information (such as logP) in the future. The database schema is simple, consisting of the one-to-one Shape Signatures table that indexes each descriptor by ZINC compound code and numerical index.

### Fast prefiltering

Individual Shape Signature comparisons are fast, requiring about 100 μs to complete. However, with the introduction of the fragment-based approach, the comparison of two molecules may involve dozens of mappings, depending on the number of fragments and their topology. For the simplest (yet very common case) of linear arrangements of fragments in both query and target, the number of mappings *M(q,t)* is easy to compute:

4 $$M(q,t) = mn + 2\left[ {\sum\limits_{k = 2}^{\begin{subarray}{l} k \le m, \\ k \le n \end{subarray} } {(m - k + 1)(n - k + 1)} } \right]$$

Here, *m* and *n* are the number of fragments in query and target respectively; the first term is the number of mappings that link single fragments, the second term counts mappings involving 2 or more links *k* between query and target, with the assumption that mapped groups of fragments are contiguous. (The factor of 2 in Eq. 4 accounts for the fact that mappings involving two or more links have two possible orientations.) A comparison of two molecules that each comprises five fragments then implies 85 mappings. While we have not accumulated statistics of fragment counts in ZINC, it is clear that the introduction of fragment comparisons will increase search times by an order of magnitude or more.

Fortunately, it is easy to efficiently prefilter the database by quickly identifying a lower bound on the fragment-based distance between two compounds. We begin by extracting from the full Shape Signatures descriptor for a library molecule the 1D (shape-only) components for the fragments, along with the segment counts for these, and storing the information in a compact binary format. (Fragment connectivity information is ignored.) Binary databases of these reduced descriptors are assembled for the compounds in each library, and these databases are small enough to be maintained as shared memory resources on our server. A shared resource is accessible to any running process, and reading data from a memory resource is very fast (since no disk access is involved). The binary shared resource for a target library is created at the same time that the corresponding Shape Signatures descriptors are imported to the local MySQL database.

When comparing a query against a reduced target descriptor, the best score for each query fragment is found using any available fragment in the target, irrespective of fragment connectivity (which is ignored when prefiltering), and with no limit on the number of times a target fragment can be used. A weighted sum of the optimal fragment–fragment scores is assembled, using the same approach implemented for full fragment-based scoring (Eq. 2). The prefilter score thus presents a lower bound for the score of a maximal match between query and target. The fast prefilter is used to generate an initial hit list of 20-30,000 compounds, which are then subject to detailed comparison by the full mapping algorithm. It is the full mapped matches which are presented to the user.

While the prefilter could potentially screen out some useful matches (since substructure matches can evince a better score than mappings involving all fragments), this is mitigated by retaining a large prefilter list (5–10 X the final number of hits to be retained). We also emphasize that our mapping strategy is “greedy” by default, with maximal expansion of each seed single-fragment mapping, and that substructure matches are thus deemphasized.

While the CPU time for a query depends critically on the structure and search parameters, a typical Shape Signatures search against ZINC for a drug-like molecule takes about 15 min of CPU time for a 2.8 GHz Intel server running the OS X operating system.

### Characteristics of fragment-based scoring

As noted above, the hit list for Novobiocin generated using the original non-fragment Shape Signatures method included many ZINC compounds that are interesting matches to the query, involving variations of its structure while retaining overall form; however, these often appear with poor rank, and are distributed at random among the hits.

Table 1 compares the ranks of the compounds in Fig. 1 as found using the original, non-fragment approach and our new method (chemical structures are shown in SM-1). The IDs of compounds judged interesting (as evincing significant structural similarity to the query) are highlighted in bold. Clearly, the fragment approach has succeeded for this subset in demoting uninteresting compounds (in fact, all but one fall out of the top 5,000 hits), and promoting interesting hits that were previously low-ranked to a high position. At the same time, the hit compounds that are essentially identical to the query remain at the top of the list, albeit with some reordering of rank position. A detailed examination of top hits reveals a clear enrichment in compounds that we deem interesting on the basis of chemical intuition (i.e. evincing variation with respect to the query while retaining significant overall similarity).

**Table 1:** Comparisons of compound hit ranks

Compound ID	Original rank	Fragment-based rank
**ZINC72693277**	1	1
**ZINC53230370**	2	3
**ZINC03831234**	3	9
**ZINC14879999**	4	2
**ZINC60021800**	5	5
**ZINC03831232**	6	8
**ZINC17611745**	7	7
**ZINC17611750**	8	11
**ZINC76945632**	9	13
ZINC54006905	21	nr*
ZINC19234908	22	nr*
ZINC09683120	23	nr*
ZINC04797250	24	4,556
ZINC69266020	25	nr*
ZINC40158598	26	nr*
**ZINC04102306**	730	20
**ZINC43682802**	783	22
**ZINC43682806**	183	24
**ZINC64447983**	2,041	26
**ZINC49654426**	1,444	29
**ZINC26723060**	6,118	33

(*) not ranked in top 5,000 hits

The apparent randomness of rank order using the original non-fragment approach is illuminated by examining the distribution of scores. For this purpose it is useful to apply a log transform to the distance scores, as these are compressed in a narrow range (0–2)—at the same time we recast this as a similarity measure, with increasing positive score representing smaller distance between descriptors:

5 $$T(s) = 1 - \log_{2} (s)$$

**Fig. 8:**
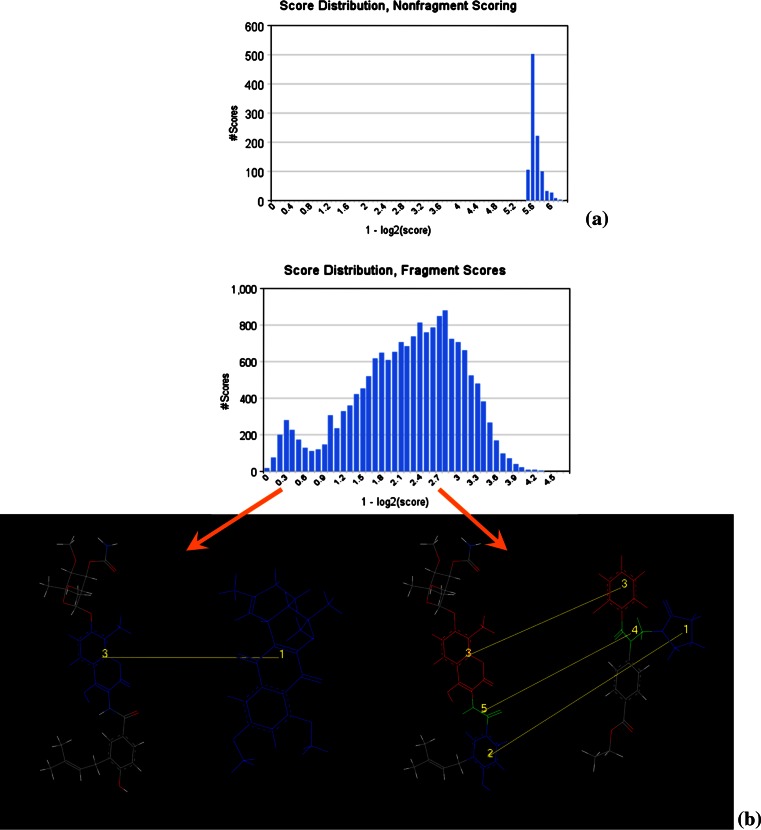
Comparison of transformed score distributions (Eq. 5) for non-fragment (**a**) and fragment-based **b** Shape Signatures. In (**b**) some fragment matches typical of poorer scores are illustrated, with the query (Novobiocin) on the left, the matching ZINC compound on the right; these typically involve partial matches between query and target compound

The transformed score *T(s)* has a lower bound of zero (corresponding to a maximal inter-descriptor distance of 2.), and tends to positive infinity as the raw score approaches zero. (Since the generation of Shape Signatures involves a stochastic process (ray-tracing), the raw (distance) score for a comparison will usually not be identical to zero even if the chemical structures and atomic coordinates for query and target match exactly.) Figure 8 compares transformed score distributions for Novobiocin used as a query against ZINC, for both the original and fragment-based approaches. In the absence of fragment scoring, the distribution is sharply-peaked, suggesting that compounds have been screened on the basis of rough size similarity with little selectivity as to the details of shape; in contrast, the introduction of fragment-based scoring dramatically broadens the distribution, and a sampling of structures from various portions of the distribution confirms that selectivity is greatly enhanced. Structures selected from the top tail of the distribution evince close similarity to the query (similar to the examples in Fig. 1d), those taken from the middle typically represent rough substructure matches, while compounds selected from the low end generally represent poor matches between diverse fused ring systems (which are treated as single fragments).

## Validation study

Our approach to formulating an extensive validation for fragment-based Shape Signatures focuses mainly on it’s ability to select structurally diverse compounds likely to exhibit a desired bioactivity; paradoxically, a rigorous validation based on shape comparison alone is a secondary consideration. This is because a key feature of our approach is the ability to make good substructure matches, where the selected library compounds may evince significant *local* shape dissimilarity with respect to the query, and where a straightforward determination of hit quality becomes problematic if we focus only on *global* shape similarity. That said, we have also carried out a validation study using an independent shape comparison technique, as described below.

Thus, we mainly focused on showing the ability of Shape Signatures to select a list of hit molecules with significant enrichment in a desired biological activity. We focus on the Androgen Receptor (AR), an important drug target in Prostate Cancer (PCa) therapy [24], and one with which we have significant prior computational experience [25]. This is a target with a number of representative crystal structures available, including complexes with a variety of ligands. Moreover, the ZINC database (zinc.docking.org) includes a large selection of annotated binders for this target, and the Directory of Useful Decoys [26, 27] (DUD, dud.docking.org) provides a pool of nonbinding drug-like molecules with strong physicochemical similarity to known AR actives, serving as a source of negative controls.

We carried out three validation studies, described below, each involving a different Virtual Screening (VS) scenario. The first is primarily directed toward establishing benchmarks to evaluate the quality of Shape Signatures Hits by applying a secondary validation screen, here molecular docking using a rigid receptor. The second study expands on the first by considering a much larger population of compounds, while the third study explores the impact of taking receptor flexibility into account. Finally, we carried out a fourth study to directly assess the capability of the method in screening compounds purely on the basis of shape, by comparing against an independent state-of-the-art shape comparison tool.

### Computational details

#### Protein structure preparation and alignment

High-resolution (<1.80 Å) crystal structures of Androgen Receptor Ligand Binding Domain (ARLBD) used in the validation study (Table 2) were all downloaded from the RCSB Protein Data Bank [28] (http://www.rcsb.org) in September 2012. Structures were prepared using the Protein Preparation Wizard [29, 30] available from Schrödinger [31] (Suite 2012 for OS X). During preparation, protein receptors were aligned on the three-dimensional (3D) coordinates of PDB ID 2AM9 [32] as reference template; missing hydrogen atoms were added and molecules of water located within 5 Å of the ligand were retained. The program Prime [33–35] was used to predict missing side chains, when necessary. As for the ligands, bond orders and formal charges were adjusted before performing full Molecular Mechanics (MM) minimizations (by OPLS [36–38] 2005 force field, at 0.30 Å convergence) of AR complexes by running the *impref* utility from the Impact program [39].

**Table 2:** Ligands used as Shape Signature queries for VS of ZINC database

PDB ID	Ligand ID	Chemical structure	Ligand name	Resolution (Å)
1XOW	R18		(17β)-17-HYDROXY-17-METHYLESTRA-4,9,11-TRIEN-3-ONE	1.80
1Z95	198		R-BICATULAMIDE	1.80
2AM9	TES		TESTOSTERONE	1.64
2AX6	HFT		HYDROXYFLUTAMIDE	1.50

Crystal structures of human Androgen Receptor Ligand Binding Domain (ARLBD) used as queries for Shape Signatures Screening of the ZINC database for AR binders. Entries are listed in alphabetic order along with relative PDB identifiers, ligand identifiers, chemical structures, ligand names and resolution (Å)

#### Ligand preparation

All ligands submitted to docking simulations of Case Studies I to III were either extracted from the ZINC database or downloaded from the DUD in mol2 format and prepared using the LigPrep [40] tool from Schrödinger. Chemical structures were submitted to generation of stereoisomers and alternative ring conformations. The tool Epik [41–43] was used to produce different ionization and tautomeric states at physiological pH. Finally, energy minimizations of all structures were performed with the OPLS 2005 force field.

#### Physicochemical property filters

The “Filter” ligands protocol available through the Virtual Screening Workflow [44] was used to select chemical structures resulting from Shape Signature screening of the ZINC database (Hits) or the full set of Decoys downloaded from the DUD web-site. We used the QikProp [45] tool to estimate physicochemical properties of chemical structures before filtering by the Lipinski’s Rule-of-5 [46] (log P < 5; kDa < 5; HB donors ≤ 5; HB acceptors ≤ 10) and excluding scaffolds containing poor drug-like properties or reactive chemical moieties.

#### Molecular docking

Different crystal structures of ARLBD co-crystallized with a ligand were used as docking receptors, particularly: PDB IDs 2AM9 for Single Conformation Rigid-Receptor Docking (Case Studies I and II); PDB IDs 2AM9, 1XOW [47], 1Z95 [48] and 2AX6 [49] for Ensemble Docking of Multiple Protein Conformations (EnsD) [50] (Case Study III).

Docking areas for each receptor conformation (docking grids) were defined by choosing the default size of the enclosing box and by excluding co-crystallized ligands from the binding pockets. Simulations were all performed using Glide [51–54] with Extra-Precision (XP) Scoring Function (SF), allowing full ligands flexibility and accounting for Epik state penalties for re-scoring. XP-descriptors were generated. The OPLS 2005 force field was used to perform post-docking energy minimizations.

#### Hierarchical clustering and similarity screen

Hierarchical clustering and similarity screen were performed of both the AR Ligands and the Fragment Based Shape Signatures Hits using Canvas [31, 55]. Radial fingerprints (ECFP) were selected as binary descriptors [56]. Structure similarity was calculated using the Tanimoto coefficient. In *hierarchical clustering,* the linkage was performed using the “average method”. In *similarity screen,* the AR Ligand ZINC03814409 was used as common reference molecule to evince structural similarity. (Since ZINC03814409 was not included in the original top 80 VS hits, it was added to this set before the calculation.)

#### Phase Shape screening

Phase Shape screening was performed using the program Phase [57, 58]. Descriptors were generated applying the pure volume scoring (“Shape Sim Pure”) and compared to 1D Shape Signatures. Ligands were extracted from the ZINC database or downloaded from the DUD. Existing conformers were kept and additional conformers were generated (up to 100). Multiple conformers (up to 10) per rotatable bond were retained. During conformational searches, amide bonds were allowed to freely rotate.

### Case Study I: Comparison of Shape Signatures Hits to AR Ligands, Decoys and ZINC drug-like compounds by rigid receptor docking

Here we tested Fragment-Based Shape Signatures for their ability to provide screening hits that are likely to be tight binders to the ARLBD, by virtue of shape similarity to known AR ligands (i.e. compounds that co-crystallize with ARLBD). Specifically, we obtained docking scores of Shape Signatures Hits by rigid receptor docking against a single AR conformation, and compared these to results for positive and negative control compounds. We are especially interested in the enrichment of molecules with “good to excellent docking scores” among the Shape Signatures compounds.

#### Virtual screening by Shape Signatures

**Fig. 9:**
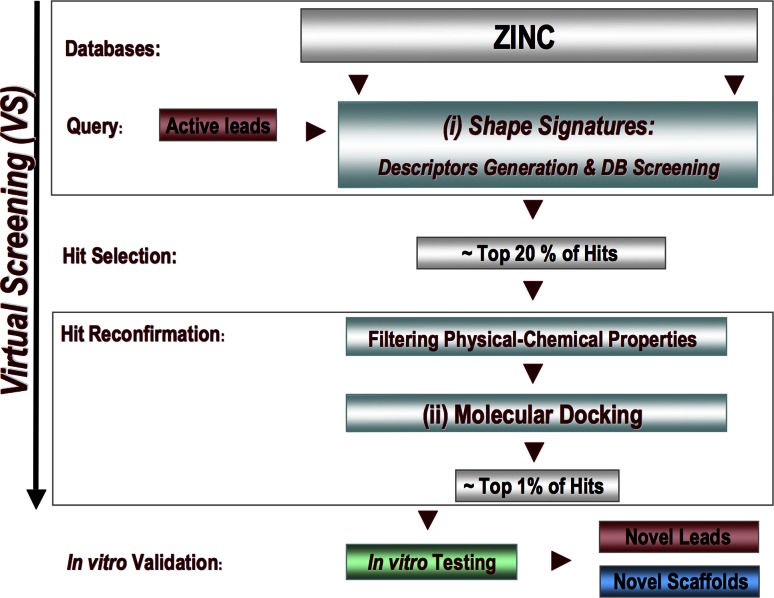
Virtual screening (VS) of the ZINC database (v. 11, ~11 millions of chemical structures). VS was performed using a two-step procedure: (1) *Ligand*-based screening by Shape Signatures; (2) *Receptor*-based confirmation of top-scoring hits (~20 %) by molecular docking simulations upon filtering by physicochemical properties according to Lipinski Rule-of-5. Typically, a minimum of 1 % of reconfirmed hits with high drug design potential is prioritized for purchase (or synthesis) upon visual inspection of binding modes and/or approval by expert medicinal chemists before in vitro testing. Both *new* *analogs* of existing lead compounds or *novel* *chemical scaffolds* are likely to be obtained

We performed iterative screening of the ZINC database using a set of four structurally different ligands used as query molecules (Table 2) in Shape Signatures searches (Fig. 9).

It is well known that the selection of query molecules is a critical aspect in ligand-based VS, particularly in the case of ligands producing significant induced-fit effects in a receptor upon binding. Recent efforts have been expended to analyze, classify and retrieve sets of “diverse” binding sites for the same protein when accommodating structurally different chemical classes of binders, based on automatic clustering of volume overlaps [59].

In a recent work [25], we have performed accurate structural analysis of a number of crystal structures of the human ARLBD in complex with a diverse set of ligands, which revealed insights into AR flexibility upon binding to structurally distinct compounds. Our results suggested similarities and differences in the molecular determinants responsible for AR binding, and were used as the foundations for selecting appropriate ligands for Shape Signatures searches. All queries were extracted from publicly available, high quality crystal structures of the human ARLBD (resolution < 1.80 Å; Table 2): 2AM9, 1XOW, 1Z95 and 2AX6. To account for the structural diversity of AR binders, we selected two Steroid (SL; 2AM9 and 1XOW) and two Non-Steroid Ligands (NSL; 1Z95 and 2AX6). Both NSLs behave as AR antagonists of the wild-type AR, while exerting agonistic properties against mutant ARs.

All co-crystal structures were downloaded from RCSB Protein Data Bank in September 2012 and prepared following the standard workflow implemented in the Protein Preparation Wizard. Ligands were extracted from their respective receptors and saved in a Shape Signatures compatible format (mol2). For each query (4 known AR binders), 1D Shape Signature descriptors (shape only) were used to individually search all ZINC compounds by comparisons with their previously generated Shape Signatures descriptors. (Details in “Methods”.)

Hits were excluded from the final lists if more than 25 % of query or target were unused when forming a mapped comparison (see “Methods”). For each run, a total of 20,000 ZINC molecules were pre-filtered, and from these up to 5,000 compounds were selected by detailed mapped comparison to form the final hit list for each query. The union of the four hit lists provided a total of 15,338 compounds (including duplicates), which were merged to produce a non-redundant list of 14,032 molecules.

#### Benchmark compound sets

As a metric for assessing the performance of VS by Shape Signatures, we estimated ligand enrichment of top-ranking screening hits (assessed by detailed molecular docking) versus three sets of reference control compounds, thus comparing docking score distributions for a total of four benchmark sets (Table 3):

**Table 3:** Case I statistics: Shape Signatures screening and rigid receptor docking

	ShSig. Hits	AR Ligands	AR Decoys	ZINC Sel.
# ZINC IDs	80	79	80	80
# Docked structures	664	478	253	237
# Best poses	75	67	74	35
Best score (kcal/mol)	−10.7	−11.0	−9.90	−7.95
Worst score (kcal/mol)	−4.24	−5.66	−3.05	−0.730

For each benchmark set used in Validation Case Study I (Sh-Sig Hits: Shape Signatures Hits; AR Ligands & Decoys: from the Directory of Useful Decoys DUD; ZINC Sel: Random Selection from the “Drug like” Subset), the number of ZINC compounds selected for validation by molecular docking simulations are reported (# ZINC IDs), along with the actual numbers of structures obtained by Ligands Preparation (# Docked Structures). As for docking results, the number of ZINC IDs with at least one binding mode (# Best poses) is reported along with docking score ranges by Glide XP Scoring Function

1. *Shape Signatures Hits* (“test set”), comprising 80 compounds selected from the four Shape Signature searches (top 20 for each query ligand).
2. *AR Ligands* including all 79 annotated AR binders (“true positives”) downloaded from the DUD.
3. *AR Decoys Subset*, consisting of 80 compounds randomly selected from the full set of AR Decoys (2,854 compounds) downloaded from the DUD. By definition, AR Decoys show physicochemical properties similar to those of AR ligands but with dissimilar topologies. Therefore, they are unlikely to be binders.
4. *ZINC Random Selection* from the “Drug like” Subset, including 80 unique compounds that were downloaded by choosing the “Clean Drug-like subset” (query performed in September 2012), then browsing for sample molecules. A total of 136 compounds were downloaded, 80 of which were randomly picked. All these compounds show physicochemical properties according to Lipinski’s Rule-of-5 and, according to their selection process, they are improbable AR binders.

When comparing against the test set of Shape Signature Hits, we defined AR Ligands as *positive* controls, while AR Decoys and the ZINC Random Selection Set were assigned as *negative* controls.

#### Molecular docking

Benchmark compounds were prepared for docking simulations according to the ligands preparation procedure using LigPrep. Total numbers of structures obtained after preparation are reported in Table 3. Docking simulations were performed against the crystal structure of the human ARLBD (PDB ID 2AM9) as rigid receptor, using Glide with XP SF. (Refer to “Computational Details” in the “Methods” section.)

Post-docking processing was conducted using the “Select Top Poses” script, available from Schrödinger [31]. For each compound, best binding modes were selected according to docking score (kcal/mol).

**Fig. 10:**
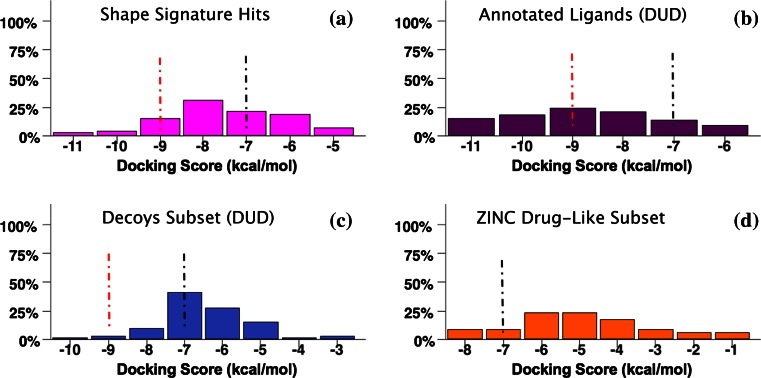
Case Study I Results. Frequency distributions of Glide XP docking scores (kcal/mol) for benchmark set compounds obtained by Rigid-Receptor Docking of Single Protein Conformation: **a** Shape Signature Hits (80 starting compounds, 75 individual binding modes); **b** annotated AR Ligands available at DUD (79 starting compounds, 67 individual binding modes); **c** random-selected subset of AR Decoys available at DUD (80 docked compounds, 74 individual binding modes) and **d** subset of ZINC Drug-like compounds random selected from the “clean compounds library” (80 starting compounds, 35 individual binding modes obtained). Two docking score thresholds are indicated: score (***s***) ≤ 7 kcal/mol, suggesting hits (but not leads, in which case more stringent criteria should be satisfied) as *possibly* true positive, on condition that further validation are capable of confirming; ***s*** ≤ 9 kcal/mol, excellent score, meaning hits having high chances of being *true* AR binders

The final step is to evaluate the likelihood of having identified a true positive from the docking score, which requires establishing thresholds. Defining such thresholds for docking scores can be a non-trivial task, since the significance of scores depends critically on the target protein. In our case, the main criterion to define hit significance was the docking scores distribution of the known AR Ligands (Fig. 10). A secondary consideration was the accepted definitions of significant score ranges as described in the literature for Glide [52–54] XP SF. These two principles were in fact in good agreement, and led to the following docking score (***s***) definitions:

- ***s*** ≤ −9 kcal/mol: excellent docking score, hits have high chances of being true AR binders.
- ***s*** ≤ −7 kcal/mol: about 20 % of AR Ligands have approximately this docking score (as shown in “Results”), which was set as a putative threshold to define virtual screening hits, and possible true positives. (A score of −6.5 kcal/mol is the value below which fall 98.6 % of best binding modes of AR Ligands when docked against multiple receptors; however, to limit the false positive rate, we chose a more stringent cutoff of −7 kcal/mol.)
−6 < ***s*** < − 6.5 kcal/mol: generally regarded as poor scores, where SFs have lost their ability to clearly distinguish between true positives and false negatives. In fact, few or no AR true Ligands lie in this score range (3.8 % in Case Study I and 0 % in Case Study III; discussed in “Results”), which was named “the grey zone”, since no conclusions can be readily drawn;
- ***s*** ≥ − 6 kcal/mol: low chances of being true positives in the design of AR binders.

### Case Study II: Virtual screening by Shape Signatures, reconfirmation by rigid receptor docking and comparison to Decoys Set

Using the scoring thresholds developed and successfully applied for a relatively small set of positive and negative controls (Case Study I), our next step was to expand our scope and assess the performance of Shape Signatures in the context of a larger set of negative controls, here the *entire* set of AR Decoys available at DUD.

Virtual screening by Shape Signatures of the ZINC database was performed as described in the corresponding section of Case Study I, however for the present case an expanded selection of top hits was used to match the increased size of the negative control set.

#### Benchmark compound sets

We estimated ligand enrichment of top-ranking Shape Signature Hits versus the entire set of AR Decoys available at the DUD. Our sets for benchmark calculations include (Table 4):

**Table 4:** Case II Study Statistics: Shape Signatures screening and rigid receptor docking

	ShSig. Hits	AR Decoys
# ZINC IDs	2,854	2,854
# Structures prepared	25,532	11,793
# Docked structures	22,880	10,848
# Best poses	2,101	2,373
Best score (kcal/mol)	−10.8	−10.9
Worst score (kcal/mol)	1.85	0.771

^***a***^For each benchmark set used in Validation Case Study II (Sh-Sig Hits: Shape Signatures Hits; AR Decoys: from the Directory of Useful Decoys DUD), the number of ZINC compounds selected for validation by molecular docking simulations are reported (# ZINC IDs), along with the actual numbers of structures obtained by Ligands Preparation (# Structures Prepared), filtering by physicochemical properties and elimination of unwanted moieties, like reactive substructures (# Docked Structures). As for docking results, the number of ZINC IDs with at least one binding mode (# Best poses) is reported along with docking score ranges by Glide XP Scoring Function. AR Decoys were downloaded from the Directory of Useful Decoys DUD

1. *Shape Signatures Hits*, comprising 2,854 ZINC compounds selected by choosing top-ranking hits from the merged Shape Signature searches.
2. *AR Decoys*, including the full set of 2,854 structures showing physicochemical properties similar to those of AR Ligands but with dissimilar topologies. Therefore, they are unlikely to be binders.

All chemical structures were prepared following the Ligands Preparation procedure (described in the “Computational Details” of the “Methods” section) to provide final lists of 25,532 and 11,793 individual structures in case (1) and (2), respectively (Table 4).

It should be noted that although the number of hits selected in this study for validation by molecular docking was established for consistency with AR Decoys (2,854 each set), it represents about 20 % of the total compounds (non-redundant list) retrieved by Shape Signatures (14,032), which is a reasonable fraction of compounds to choose for computational reconfirmation in a *real*-world *virtual* screening campaign. In fact, Case Study II follows our expected workflow for Shape Signatures, with this ligand-based method used as a primary screen, and with a significant fraction of the top-ranking compounds validated by a second, more computation-intensive approach, such as detailed molecular docking (Fig. 9).

Before submitting these ligands to molecular docking simulations, their drug-likeness was assessed by applying molecular filters to discard molecules that do not comply with Lipinski’s Rule-of-5 or contain reactive chemical moieties. To do so, we used the “Filter” ligands protocol available through the Virtual Screening Workflow (Schrödinger Suite 2012) to calculate their physicochemical properties by QikProp and remove reactive chemical structures. Thus, only final sets of “drug-like” compounds (22,880 hit and 10,848 decoy structures) were submitted to docking simulations (Table 4).

#### Molecular docking

Molecular docking simulations of both Shape Signatures Hits and AR Decoys were performed against a single AR conformation, as described in the corresponding section of Case Study I, and in the Computational Details of the Methods section.

### Case Study III: Comparison of Shape Signatures Hits to AR Ligands and ZINC drug-like compounds by protein ensemble docking

The first two case studies seek to establish Shape Signatures as an effective tool in virtual screening, by providing a fast first filter of very large compound collections and producing hits with desired shapes that, upon confirmation, are likely to develop into lead candidates for a particular drug target (AR in our case).

While Shape Signatures, the first step of our virtual screening implementation, is *ligand*-based, the second is *receptor*-based, namely by molecular docking. In keeping with common practice, we made our validation step as efficient as possible by docking against a single, rigid model of the target receptor. However, it is well known that ARLBD adopts significantly different conformations upon binding to various chemical classes of ligands, suggesting that the false negative rate in our first studies might be exaggerated (as alternative conformations of the receptor were ignored), leading to an underestimation of the enrichment factor attainable with the Shape Signatures approach.

To incorporate AR plasticity into a flexible receptor docking protocol, we performed Ensemble Docking of Multiple Protein Conformations (EnsD) [50]. This protocol involves individually docking Shape Signature hits and representative reference-set compounds against all the available forms of the receptor (here the four ARLBD conformations co-crystallized with the ligands used as screening queries), followed by selection of top scoring docking poses (by absolute docking score, kcal/mol) as best binding modes.

#### Benchmark compound sets

Benchmark set compounds are listed in Table 5 and include: Shape Signature Hits (80 total), AR Ligands (79 total) and ZINC Random Selection from the “Drug like” Subset (80 total), that were collected as described in the proper section of Case Study I.

**Table 5:** Case study III statistics: Shape Signatures screening and ensemble docking

	Shsig. Hits	AR Ligands	ZINC Sel.
# ZINC IDs	80	79	80
# Docked structures	664 (× 4)	478 (× 4)	237 (× 4)
# (Absolute) best poses	78	70	68
Best score (kcal/mol)	−10.7	−11.2	8.30
Worst score (kcal/mol)	−4.91	−5.66	−0.495

For each benchmark set used in Validation Case Study III (Sh-Sig Hits: Shape Signatures Hits; AR Ligands: from the Directory of Useful Decoys DUD; ZINC Sel: ZINC Random Selection from the “Drug like” Subset), the number of ZINC compounds selected for validation by molecular docking simulations are reported (# ZINC IDs), along with the actual numbers of structures obtained by Ligands Preparation that were submitted to docking against 4 receptors (# Docked Structures; × 4). As for docking results, the number of ZINC IDs with at least one binding mode (# (Absolute) best poses) is reported along with docking score ranges by Glide XP Scoring Function

#### Molecular docking simulations

Molecular docking simulations of benchmark set compounds were individually performed against an ensemble of multiple protein structures, selected to reproduce induced-fit effects of ARLBD upon binding to ligands used as Shape Signatures queries. Thus, crystal structures of 2AM9, 1XOW, 1Z95 and 2AX6 were prepared according to the Protein Preparation Wizard before using the Glide tool to generate docking grids and perform molecular docking simulations at XP level SF. (Described in “Computational details” paragraph of the “Methods” section.)

Then, for each compound set, docking results against individual protein structures were processed using the “Select Top Poses” script, available from Schrödinger platform. For each compound within a set, the best binding mode against a particular receptor was selected as *relative* best pose according to the lowest docking score (kcal/mol). Comparisons of up to four relative best poses, obtained for each compound of a particular set against multiple protein conformations, allowed selecting the lowest docking scores (kcal/mol) as *absolute* best poses.

### Case Study IV: Comparison with an independent shape screening method

#### Rescoring fragment based shape Signatures Hits by Phase Shape

Since it is not practical for us to generate Phase Shape descriptors for the >11 million compounds in our augmented ZINC database, we opted for “re-scoring” hits generated by Fragment Based Shape Signatures to enable a direct comparison of the two methodologies.

To this end, we considered hits retrieved by searching the ZINC database using Novobiocin as query ligand. This query compound choice was motivated to ensure a high order of complexity in the “compound fragmentation” stage required during the Shape Signatures generation. As a matter of fact, Novobiocin represents a challenging example of multiple fragment mappings, as shown in Fig. 3.

We used as an additional target for comparison the ‘ad hoc’ collection comprised of 79 AR Ligands and 2,854 AR Decoys from the DUD, plus 80 random selected compounds from the ZINC database (total of 3,013 individual structures), as introduced above. This set includes a number of steroidal compounds, and against this set we used Testosterone (ligand from PDB complex 2AM9) as the query. Testosterone presents a single fragment in our new Shape Signatures approach, and will lead to results in line with the original non-fragment-based implementation. This permits a comparison of efficacy of the original and new approaches for enriching compound selections solely on the basis of shape.

## Results and discussion

### Case study I

We tested the ability of fragment-based Shape Signatures to provide virtual screening hits with high potential to identify chemical classes of AR binders. To reconfirm Shape Signatures results, we calculated hit rates (number of hits within a particular range of docking score) by performing molecular docking simulations against a single AR conformation (PDB ID 2AM9). We used docking scores (kcal/mol) as a measure of ligand enrichment of compounds from the test set (top scoring Shape Signatures Hits) against positive- (AR Ligands) negative-control compounds (AR Decoys and ZINC Drug-like Subset). Ideally, docking scores of the test set should be clearly better (more negative docking scores or higher numbers of good scoring hits) than those obtained from any of the negative controls. In addition, it would be very desirable to obtain hit rates of compounds retrieved by Shape Signatures searches that are “closer” to the positive control than any negative controls. (Important to mention that *all* the compounds in the AR Ligands set are experimentally proven AR binders, while VS hits are typically *not* expected to be true positives after experimental verification.)

Distributions of Glide XP docking scores (kcal/mol) of Shape Signatures Hits, AR Ligands (positive control), Decoys and ZINC Drug-Like Subsets (negative controls) are listed in Table 6 by counting the total number of compounds according to scoring category, using the scoring ranges defined in “Methods”. Histograms (by Canvas Schrödinger) [55] showing frequency distributions of benchmark set compounds within Glide XP score ranges (kcal/mol) are reported in Fig. 10a–d.

**Table 6:** Case Study I Results: Shape Signatures screening and rigid receptor docking

Score (kcal/mol)	Sh-Sig. Hits	AR Ligands	AR Decoys	ZINC Sel.
***s*** ≤ (−6.5)	68.8 % (55/80) 73.3 % (55/75)	77.2 % (61/79) 91.0 % (61/67)	51.3 % (41/80) 55.5 % (41/74)	7.50 % (6/80) 17.1 % (6/35)
***s*** ≤ (−7.5)	52.5 % (42/80) 56.0 % (42/75)	65.8 % (52/79) 77.6 % (52/67)	13.8 % (11/80) 14.9 % (11/74)	3.75 % (3/80) 8.60 % (3/35)
***s*** ≤ (−8.5)	22.5 % (18/80) 24.0 % (18/75)	49.4 % (39/79) 58.2 % (39/67)	3.75 % (3/80) 4.05 % (3/74)	0 %
***s*** ≤ (−9.5)	7.50 % (6/80) 8.00 % (6/75)	29.1 % (23/79) 34.3 % (23/67)	0 %	0 %
***s*** ≤ (−10.5)	3.75 % (3/80) 4.00 % (3/75)	12.7 % (10/79) 14.9 % (10/67)	0 %	0 %
(−6.0) < ***s*** < (−6.5)	12.0 % (9/75) 12.0 % (9/75)	3.80 % (3/79) 4.48 % (3/67)	10.0 % (8/80) 10.8 % (8/74)	5.00 % (4/80) 11.43 % (4/35)
***s*** ≥ (−6.0)	13.8 % (11/80) 14.7 % (11/75)	3.80 % (3/79) 4.48 % (3/67)	31.3 % (25/80) 33.8 % (25/74)	31.3 % (25/80) 71.4 % (25/35)
***s*** ≥ (−5.0)	5.00 % (4/80) 5.33 % (4/75)	0 %	7.50 % (6/80) 8.11 % (6/74)	20.0 % (16/80) 45.7 % (16/35)
***s*** ≥ (−4.0)	0 %	0 %	2.50 % (2/80) 2.70 % (2/74)	11.25 % (9/80) 25.7 % (9/35)
***s*** ≥ (−3.0)	0 %	0 %	2.50 % (2/80) 2.70 % (2/74)	7.50 % (6/80) 17.1 % (6/35)
***s*** ≥ (−2.0)	0 %	0 %	0 %	2.50 % (2/80) 5.70 % (2/35)
No binding modes	6.25 % (5/80) 6.67 % (5/75)	15.2 % (12/79) 17.9 % (12/67)	7.50 % (6/80) 8.11 % (6/74)	56.3 % (45/80) 129 % (45/35)

Docking score distributions (Glide, XP) of benchmark set compounds (Sh-Sig Hits: Shape Signatures Hits; AR Ligands & Decoys: from the Directory of Useful Decoys DUD: ZINC Sel: ZINC Random Selection from the “Drug like” Subset) are given as percentages of compounds that meet the criteria and as absolute numbers (in parenthesis). For each set, percentages are given (1) as the number of compounds having a score above or below a particular threshold over the total number of compounds submitted to docking simulations and (2) over the total number of compounds with at least one binding mode retrieved by individual docking runs. Percentages and absolute numbers of compounds without any binding modes predicted are also reported (no binding modes)

The results met these expectations, with Shape Signatures Hits evincing a behavior much closer to true AR Ligands (positive controls) than either AR Decoys or randomly-selected drug-like ZINC compounds (negative controls). There is strong preference for Shape Signature Hits over negative controls, as reflected in higher percentages of compounds, in both the ‘good’ and ‘excellent’ docking score ranges (***s*** ≤ −7 to ***s*** ≤ −9 kcal/mol).

Furthermore, clear separations appear among frequency distributions of docking score (kcal/mol; Glide XP) of Shape Signature Hits compared against negative control sets (Fig. 10), thus confirming our hypothesis that Shape Signatures performs well in filtering large compound collections for molecules having desired shape similarity to a bioactive query, and therefore likely to constitute true positive hits against a drug target of interest.

### Case study II

With a view of identifying an expanded set of chemical compounds that might serve as AR modulators, we put in place a VS workflow based on a two-step procedure (Fig. 9): first, we performed Shape Signature screening of the ZINC database with multiple active compounds selected as searching queries; second, we reconfirmed top-ranking hits by molecular docking, but in this case with the entire AR decoy set, thus providing a more realistic screening scenario. This second study is supported by the results of Case Study I, which confirmed our choice of docking scoring ranges as measures of hit significance.

We used four distinct Shape Signature queries (Table 2) to screen version 11 of the ZINC database, which consisted of 11,080,665 compounds. A total number of 14,032 Shape Signatures hits were obtained by merging results from individual screening campaigns, selecting ~0.13 % of the initial library for computational follow-up. Of these, we selected 2,854 top-ranking hits (~20 %) that were submitted to reconfirmation by molecular docking. In the interest of docking accuracy we chose Glide XP SF and we saved overall computational time by using a rigid receptor protocol with a single AR conformation.

Overall, virtual screening by Shape Signatures produced remarkably high hit rates. As previously discussed, we considered a docking score −9 kcal/mol as a reliable threshold to select compounds having strong predicted binding (in the range of low micromolar to nanomolar IC_50_ values). We calculated hit rates (%) of Shape Signatures screening (Table 7) in two ways: as the total number of hits with a particular docking score, in this case ***s*** ≤ −9 kcal/mol (135 compounds), (1) over the total number of structures obtaining at least one binding mode by docking simulations (2,101), that is 6.4 %, and (2) over the total number of Shape Signatures Hits (2,854) initially selected for docking calculations, i.e. 4.7 %. (Hit rates obtained at additional score values are reported in Table 7.)

**Table 7:** Case Study II Results: Shape Signatures screening and rigid receptor docking

Score (kcal/mol)	Sh-Sig. hits	AR decoys
***s*** ≤ (−6.5)	43.0 % (1,227/2,854) 58.4 % (1,227/2,101)	35.8 % (1,022/2,854) 43.1 % (1,022/2,373)
***s*** ≤ (−7.5)	23.4 % (668/2,854) 31.8 % (668/2,101)	11.5 % (327/2,854) 13.8 % (327/2,373)
***s*** ≤ (−8.5)	8.72 % (249/2,854) 11.9 % (249/2,101)	4.80 % (137/2,854) 5.77 % (137/2,373)
***s*** ≤ (−9.5)	2.59 % (74/2,854) 3.52 % (74/2,101)	2.10 % (60/2,854) 2.53 % (60/2,373)
***s*** ≤ (−10.5)	0.140 % (4/2,854) 0.190 % (4/2,101)	0.140 % (4/2,854) 0.169 % (4/2,373)
(−6.0) < ***s*** < (−6.5)	6.48 % (185/2,854) 8.81 % (185/2,101)	10.4 % (296/2,854) 12.5 % (296/2,373)
***s*** ≥ (−6.0)	24.1 % (688/2,854) 32.7 % (688/2,101)	37.0 % (1,055/2,854) 44.5 % (1,055/2,373)
***s*** ≥ (−5.0)	10.2 % (292/2,854) 13.9 % (292/2,101)	16.5 % (471/2,854) 19.8 % (471/2,373)
***s*** ≥ (−4.0)	5.32 % (152/2,854) 7.23 % (152/2,101)	6.66 % (190/2,854) 8.00 % (190/2,373)
***s*** ≥ (−3.0)	2.14 % (61/2,854) 2.90 % (61/2,101)	2.56 % (73/2,854) 3.08 % (73/2,373)
***s*** ≥ (−2.0)	1.01 % (29/2,854) 1.38 % (29/2,101)	0.876 % (25/2,854) 1.05 % (25/2,373)
No binding modes	26.4 % (753/2,854) 35.9 % (753/2,101)	16.9 % (481/2,854) 20.3 % (481/2,373)

Docking score distributions (Glide, XP) of benchmark set compounds (Sh-Sig Hits: Shape Signatures Hits; AR Decoys: from the Directory of Useful Decoys DUD) are given as percentages of compounds that meet the criteria and as absolute numbers (in parenthesis). For each set, percentages are given as the number of compounds having a score above or below a particular threshold over (1) the total number of compounds submitted to docking simulations and (2) over the total number of compounds with at least one binding mode retrieved by individual docking runs. Percentages and absolute numbers of compounds without any binding modes predicted are also reported (No Binding Modes)

As for the comparison between Hits and Decoys it was not surprising that in significantly increasing the number of top-ranking hits submitted for reconfirmation by docking (from ~0.6 % or 80/14,032 in Case Study I to 20 % or 2,854/14,032 in Case Study II) that we observed slightly lower fractions of compounds with good to excellent docking scores (Table 6 versus Table 7). This was not unexpected, since we included a much larger proportion of compounds whose Shape Signatures were less similar to those of the active query molecules. Nevertheless, the distribution of Shape Signatures hits clearly outperforms that obtained by the tested Decoys (Table 7), by always reporting higher numbers of compounds in the desired docking score ranges (***s*** ≤ −7 kcal/mol and ***s*** ≤ −9 kcal/mol) as well as having significantly lower numbers of compounds that populate less desirable score values (***s*** > −7 kcal/mol).

**Fig. 11:**
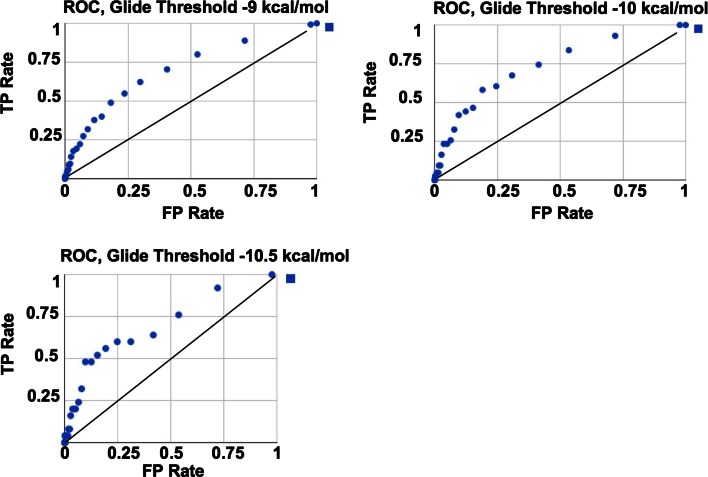
Case Study II Results. ROC curves for Shape Signatures Hits obtained by VS of the ZINC database for AR antagonists are obtained by setting progressive thresholds in the Shape Signatures score used to demarcate “predicted” positives from negatives, and with a fixed docking score benchmark used to label “true” positives and negatives. Molecular docking simulations were performed by single receptor conformation of ARLBD

In addition, with access to data for a much larger population of molecules, we were able to generate Receiver Operating Characteristic (ROC) curves for Shape Signatures Hits (Fig. 11). The raw data for the plots was generated by a custom Python script which computed true- and false-positive rates, using the Shape Signatures score as the variable threshold parameter to demarcate predicted actives from inactives in the hit list, and with a fixed Glide docking score used as a the benchmark to label compounds as positive or negative. The ROC curves, which visually confirm the performance of Shape Signatures as a binary classifier, are relatively insensitive to choice of docking score benchmark, provided the threshold is within 1 kcal/mol of our existing standard of −9 kcal/mol.

Interestingly, we conclude by observing that trends in the hit-rates (Table 7) agree with those obtained in Case Study I (Table 6), and this is a good indication that Shape Signatures performances estimated for the small compound-sets in Case Study I (~80 compounds each) are, with a close approximation, confirmed and can be easily applied to a larger set of screening compounds.

### Case study III

Protein flexibility is a critical aspect of molecular recognition, and conformational changes that occur upon binding are responsible for induced fit effects that are crucial in improving receptor/ligand affinity [60]. In a previous study [25], to deal with ARLBD plasticity, we developed a flexible receptor docking protocol based on induced fit docking [61–64] (IFD by Schrödinger, Suite 2010 on Linux Platform) to successfully reproduce geometries of complexes of known ligands and to predicting binding modes for novel compounds. We have demonstrated that IFD succeeds in “blind ligand docking”, where no structural data is available and only chemical activities of compounds against the drug target of interest are known. Here, however, our main goal is not to explore new receptor conformations adopted upon binding, but to evaluate molecules with *shapes* supposed to be *complementary* to a particular drug target by docking them into their matching protein structures. Thus, we performed Ensemble Docking (EnsD) of investigated molecules against the collection of all AR structures from which the four ligands, used as Shape Signature queries, were previously extracted.

**Fig. 12:**
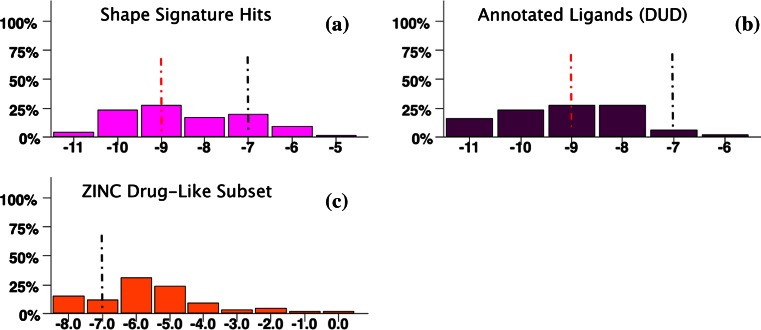
Case Study III Results. Frequency distributions of Glide XP docking scores (kcal/mol) for benchmark set compounds obtained by Ensemble Docking of Multiple Protein Conformations: **a** Shape Signature Hits (80 starting compounds, 78 individual binding modes); **b** annotated AR Ligands available at DUD (79 starting compounds, 70 individual binding modes) and **c** subset of ZINC Drug-like compounds random selected from the “clean compounds library” (80 starting compounds, 68 individual binding modes obtained). Two docking score thresholds are indicated: score (***s***) ≤ 7 kcal/mol, suggesting hits (but not leads, in which case more stringent criteria should be satisfied) as *possibly* true positive, on condition that further validation are capable of confirming; ***s*** ≤ 9 kcal/mol, excellent score, meaning hits having high chances of being *true* AR binders

EnsD results for the benchmark set-compounds, including Shape Signature Hits, AR Ligands and ZINC Drug-like Set, are reported in Table 8 and Fig. 12 (by Canvas). We find that EnsD outperforms the prediction of binding modes generated against single ARLBD, especially in the cases of Shape Signature Hits and, to a lesser degree, AR Ligands (Table 8 versus Table 6 and Fig. 12 versus Fig. 10). This is not surprising, as four dissimilar ligands, each representing a structurally distinct target, were used as queries against ZINC. By providing all of the target structures as potential binding partners, we more fully explore the range of available docking modes, albeit at greater computational expense.

**Table 8:** Case Study III Results: Shape Signatures screening and ensemble docking

Score (kcal/mol)	Sh-Sig. Hits	AR Ligands	ZINC Sel.
***s*** ≤ (−6.5)	87.5 % (70/80) 89.7 % (70/78)	87.4 % (69/79) 98.6 % (69/70)	22.5 % (18/80) 26.5 % (18/68)
***s*** ≤ (−7.5)	71.8 % (59/80) 75.6 % (59/78)	82.3 % (65/79) 92.3 % (65/70)	12.5 % (10/80) 14.7 % (10/68)
***s*** ≤ (−8.5)	53.8 % (43/80) 55.1 % (43/78)	59.5 % (47/79) 67.1 % (47/70)	0 %
***s*** ≤ (−9.5)	26.3 % (21/80) 26.9 % (21/78)	35.4 % (28/79) 40.0 % (28/70)	0 %
***s*** ≤ (−10.5)	5.00 % (4/80) 5.13 % (4/78)	13.9 % (11/79) 15.71 % (11/70)	0 %
−6.0 < ***s*** < − 6.5	7.50 % (6/80) 7.69 % (6/78)	0 %	15.0 % (12/80) 17.6 % (12/68)
***s*** ≥ (−6.0)	2.50 % (2/80) 2.56 % (2/78)	1.27 % (1/79) 1.28 % (1/70)	47.5 % (38/80) 55.9 % (38/68)
***s*** ≥ (−5.0)	1.25 % (1/80) 1.28 % (1/78)	0 %	27.5 % (22/80) 32.4 % (22/68)
***s*** ≥ (−4.0)	0 %	0 %	11.3 % (9/80) 13.2 % (9/68)
***s*** ≥ (−3.0)	0 %	0 %	8.75 % (7/80) 10.3 % (7/68)
***s*** ≥ (−2.0)	0 %	0 %	5.00 % (4/80) 5.88 % (4/68)
No Binding Modes	2.50 % (2/80) 2.56 % (2/78)	12.7 % (10/79) 14.3 % (10/70)	15 % (12/80) 17.6 % (12/68)

Docking score distributions (Glide, XP) of benchmark set compounds (Sh-Sig Hits: Shape Signatures Hits; AR Ligands: from the Directory of Useful Decoys DUD: ZINC Sel: ZINC Random Selection from the “Drug like” Subset) are given as percentages of compounds that meet the criteria and as absolute numbers (in parenthesis). For each set, percentages are given as the number of compounds having a score above or below a particular threshold over (1) the total number of compounds submitted to docking simulations and (2) over the total number of compounds with at least one binding mode retrieved by individual docking runs. Percentages and absolute numbers of compounds without any binding modes predicted are also reported (no binding modes)

Comparison between frequency distributions of docking scores (by Canvas) for Shape Signatures Hits and AR Ligands (Table 8; Fig. 12a, b, respectively) show a striking similarity: in both cases the majority of compounds fall below the −7 kcal/mol threshold, while comparable fractions of chemical structures score below the −9 kcal/mol, suggesting excellent binding affinity. In contrast, docking score distributions of compounds in the ZINC Drug-like Subset (Table 8; Fig. 12c) show a totally different profile, with nearly all chemicals scoring above the −7 kcal/mol threshold, and neither of them being better than −8.3 kcal/mol (for comparison, best scoring AR Ligands and Shape Signature Hits are −11.2 kcal/mol and −10.7 kcal/mol, respectively).

### Analysis of top scoring Fragment Based Shape Signatures Hits

The primary goal of this work was to establish Fragment Based Shape Signatures as a powerful approach in VS, particularly suitable in scaffold hopping. In this regard, we provide a description of the “top 80” screening hits, as representative results of the entire VS campaign.

We primarily focus on describing the general features of relevant scaffolds retrieved, likewise on identifying specific patterns of substituents, regardless of the chemical moiety they decorate. In particular, we seek to determine whether Fragment Based Shape Signatures is able to “fish out” compounds simultaneously exhibiting *shape similarity* and *chemical diversity*.

### “Shape similarity” by hierarchical clustering

**Fig. 13:**
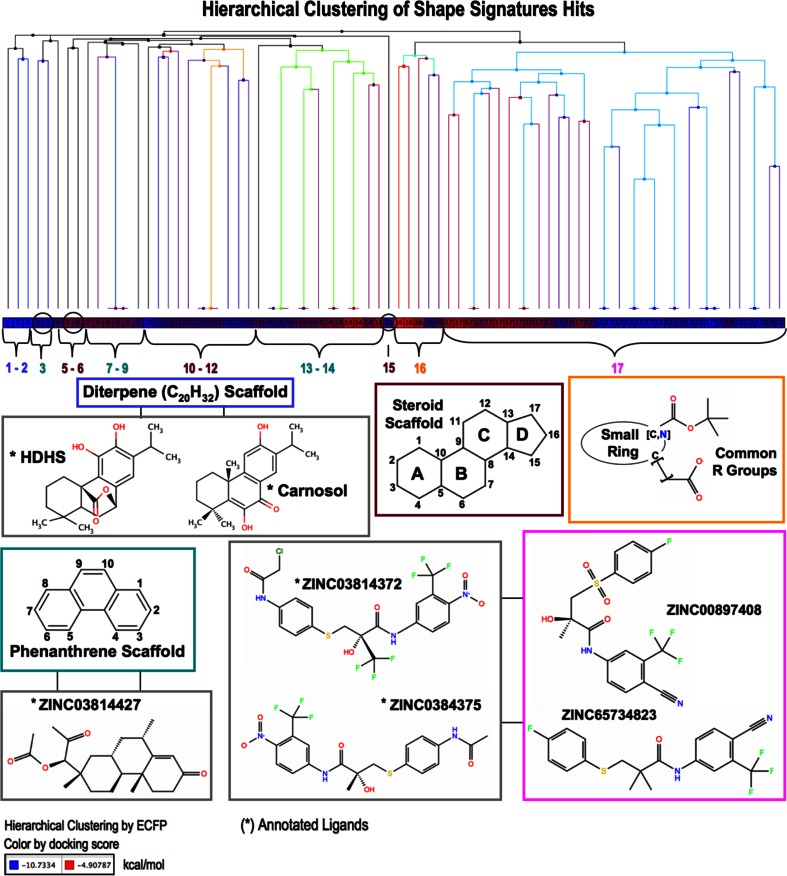
Hierarchical Clustering of Hits collected by Fragment Based Shape Signatures. Clustering by ECFP, coloring by docking score (kcal/mol). As reference compounds, known AR binders are marked with (*asterisk*) and included in gray boxes connected to the respective clusters. *Boxes* containing structures are *colored* according to cluster numbers

We performed “hierarchical clustering” of the top 80 Fragment Based Shape Signatures Hits (description in the “Computational Details” section), which grouped in 17 clusters of different size, the largest comprising 34 molecules (cluster 17). A dendrogram of the hierarchical classification is shown in Fig. 13. The plot is color-coded by docking score (by EnsD), ranging from good (−10.7 kcal/mol; in blue) to bad (−4.91 kcal/mol; in red) predicted binding.

#### Steroid scaffold

Not unexpectedly, our screening picked a significant number of structures containing the steroid scaffold (Fig. 13, bordeaux box). Although they all shared a common core, many of them introduced non-obvious variations in positions, such as C-3 on ring A and/or C-17 on ring D, known to bear the molecular determinants responsible for AR binding. These structures populated clusters 5–6, 10–12, and 15.

An important aspect in VS is the ability of the screening approach to succeed in scaffold replacement. Thus, we shifted our attention on hits bearing chemical moieties *other* *than* the parent steroid core.

#### Diterpene scaffold

One chemical class of interest was represented by hits containing the diterpene scaffold, which populated clusters 1 and 2 (blue box). Interestingly, all diterpene derivatives identified by VS showed consistent docking modes with scores up to −10.7 kcal/mol, indicating strong predicted binding.

In support of these findings, a number of natural products belonging to this chemical class have recently gained popularity for their ability to modulate the AR, and for their potential use as chemotherapeutic agents in the treatment of PCa. To cite a few examples, the dietary diterpene compound carnosol was found to serve as AR antagonist, and to inhibit the growth of PCa cells as well as to reduce tumor formation (by 36 %) in Xenograft mice [65]. In addition, a number of diterpene derivatives isolated from *Cryptomeria japonica* were shown to inhibit AR in PCa cells [66]. One of them, the abietane diterpene HDHS was reported to also suppress tumor growth in vivo through antiproliferation and proapoptosis [67].

#### Phenanthrene scaffold

Another interesting case, derivatives of the phenanthrene ring (green box), decorated with a variety of different substituents, represented a very recurrent chemical moiety in the “top 80” hit list (clusters 3, 7, 8, 9, 13, 14). Many of these structures reported a good predicted binding (up to −8.20 kcal/mol). Encouraged by these results, we searched the list of 2,854 Fragment Based Shape Signatures hits and we found a number of additional derivatives of the phenanthrene ring, some evincing very good docking scores.

We point out that some phenanthrene derivatives are known to serve as AR binders, and we (again) took two examples from the literature in support of our findings. *First*, the phenentren-yl derivative ZINC03814427 (connected gray box) is one of the compounds enclosed in the AR Ligand set from the DUD (our positive control). *Second*, we found in the current version of the ZINC database (version 12) the phenantrenone derivative ZINC13473932 (not included in version 11 used in the screening), proposed by researchers at Pfizer as a member of a novel chemical class of glucocorticoid receptor antagonists with moderate binding affinity for human AR [68].

Although our VS did not place the specific compounds cited from the literature at the very top of the hit lists, their active scaffold was retrieved as a *high*-*ranking* chemical moiety, providing a strong indication as to the remarkable scaffold hopping ability of Fragment Based Shape Signatures. This is especially meaningful considering that molecules belonging to this class were fished out in the top 80 hits from screening multiple times the entire ZINC database (11 millions of compounds).

#### Common substituents

Another example revealing the intrinsic ability of our method for scaffold replacement is represented by the hits constituting cluster 16. Strikingly tolerant to structural diversity, this cluster grouped 5 small “fragment-like” molecules, all showing different scaffolds (orange box). As for the substitutions, three of them shared the exact same groups decorating their principal chemical moieties, while two additional compounds shared only one structural feature. One molecule in particular showed a docking score of −6.72 kcal/mol, which is very promising given its small size.

#### High diversity cluster

Highly populated, cluster 17 represents a collection of diverse structures (34 total), the majority of which are closed analogs of the non-steroidal drugs Hydroxyflutamide or Bicatulamide (Table 2). Additional structures presenting a range of variations over the scaffold rings, while maintaining the overall linear backbone of the parent molecules, were also found. A successful substructure replacement is evinced by the VS hits ZINC00897408, which is indeed the Bicatulamide drug, and the closed analog ZINC65734823 (bicatulamide sulfoxide) bearing the –SO_2_– to –S– substitution (fuchsia box). Interestingly, the latter substitution is well tolerated in that position [69], and indeed it is found in the AR Ligands ZINC0384375 and ZINC03814372 from the DUD set (connected gray box).

### “Chemical diversity” by similarity screen

One challenge in shape-based screening is to collect structures evincing a certain degree of structural diversity with respect to query molecules, usually natural ligands. Thus we used the natural AR agonist Testosterone (ZINC03814409) as the parent structure, and we performed a Similarity Screen of two compound sets, the top 80 VS hits and the AR Ligands.

**Fig. 14:**
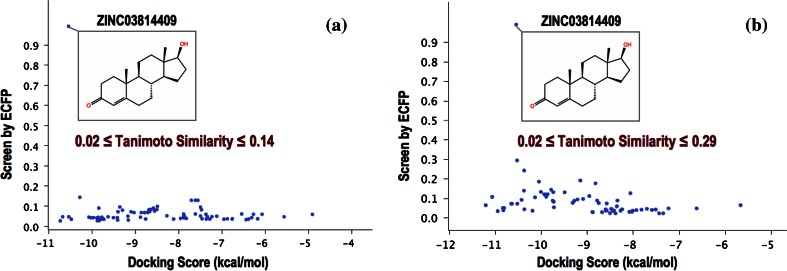
Similarity Screen. Testosterone (ZINC03814409) served as the parent structure in a similarity screen of **a** the top 80 VS hits, and **b** the AR Ligands

Overall, our analyses (Fig. 14) revealed a much lower degree of similarity to the parent steroid (by ECFP fingerprints) shared by Shape Signatures Hits (0.02 ≤ Tanimoto Similarity ≤ 0.14) than by the AR Ligands (0.02 ≤ Tanimoto Similarity ≤ 0.29). Clearly, this was an indirect indication that, on equal number of molecules, Fragment Based Shape Signatures introduced a higher degree of *chemical diversity* from the parent steroid than observed in known AR binders.

Collectively, these data demonstrate the ability of Shape Signatures to produce screening hits enriched in compounds with high drug discovery potential, and point to the utility of our method as in virtual screening and drug design.

### Case study IV

In our final study, we analyzed the utility of fragment-based Shape Signatures scores to predict similarity purely on the basis of shape (as opposed to likely bioactivity). To this end, we used the Schrödinger Phase tool, which can directly compare molecular shape using a volume overlap approach. Two query/target sets were used, Novobiocin versus the top 1,000 Shape Signatures hits found for this query in our implementation of the ZINC 11 database, and Testosterone vs. our ‘ad hoc’ collection of AR ligands and decoys described above. We visualized the results using ROC curves to compare true-positive and false-positive rates, with the Shape Signature score used as the variable control parameter. In each case, the Phase comparison was the benchmark of shape similarity, with a fixed Phase score threshold employed to assign shape similarity or dissimilarity.

**Fig. 15:**
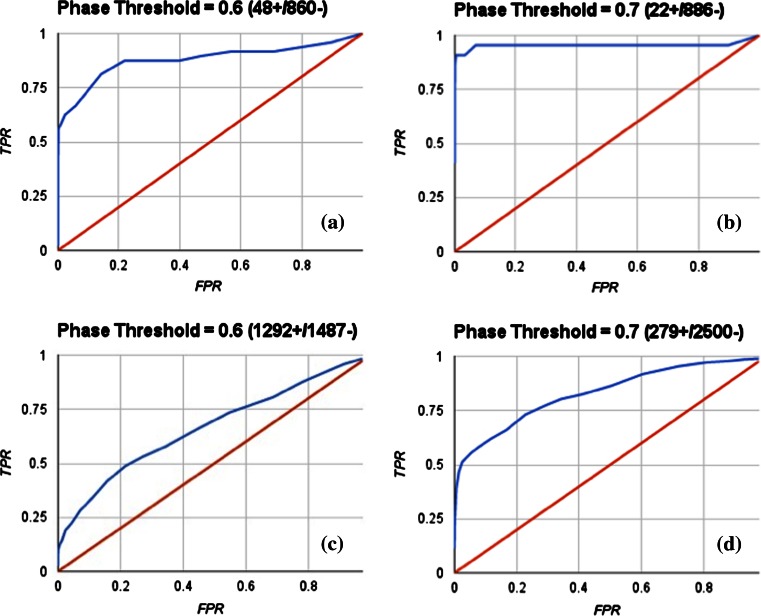
Case Study IV Results. ROC curves generated for Shape Signatures in comparison with Phase Shape. *Curves* were obtained for re-scoring Novobiocin hits (**a**) and (**b**) and for screening the ad hoc collection by Testosterone as query ligand (**c**) and (**d**). In both cases, two thresholds of Phase Shape score were set. As an example, in (**a**) the legend (48 +/860 −) means 48 positives, 860 negatives with phase threshold = 0.6

We discovered that the decomposition of the target sets into positives and negatives (shape similar or dissimilar to the query) was very sensitive to the choice of Phase score threshold. Taking a suggested threshold of 0.65 from the literature as a starting point [70], we bracketed this value with thresholds of 0.6 and 0.7. As shown in Fig. 15, the ROC curves reveal good performance for fragment-based Shape Signatures in identifying true positives, especially with the more restrictive Phase threshold of 0.7. Interestingly, the multi-fragment Novobiocin query (Fig. 15a, b) shows better performance than the single-fragment Testosterone query (Fig. 15c, d). Since the Testosterone comparison effectively mimics the previous non-fragment Shape Signatures algorithm, this provides evidence that the new fragment-based method can offer significantly-improved sensitivity and selectivity.

## Conclusions

In this work, we describe the fragment-based implementation of Shape Signatures, and demonstrate that it is a powerful tool for computer-aided drug design [8–12]. Essentially, we prove that the new methodology outperforms the older version by dramatically enhancing its selective power, while retaining all the advantages offered by the original implementation. Furthermore, we report a number of case studies used to fully assess the performance of Fagment-Based Shape Signatures as a tool in practical VS.

We conclude by summarizing three important features that emerge from these studies, which highlight Fragment-based Shape Signatures as a unique, as well as innovative methodology for computational chemistry and drug design.

*First*, Shape Signatures is a fast and effective way of screening very large compound collections. We have demonstrated that the method can produce hit lists highly enriched in interesting molecules likely to be active against a selected target. *Second*, Shape Signatures is very user-friendly approach, accessible to chemists at every level of computing expertise, and not requiring formulation of complex queries or construction of pharmacophores. One advantage of filtering databases by Shape Signatures is to assist and facilitate the process of visual inspection and application of chemical intuition by expert medicinal chemists (typically not computational specialists) by allowing them to focus on a significantly reduced number of high quality hits, which at the same time feature significant chemical diversity. *Third*, and very importantly, Shape Signatures *does not* explicitly involve chemical structure and this makes it an ideal tool allowing scaffold hopping and identification of *novel* chemical classes of drug targets modulators.

The Shape Signatures approach is available on our server (artemisdiscovery.com), and accounts will be provided for academic use at no cost, upon request.

## Electronic supplementary material

Below is the link to the electronic supplementary material.

Supplementary material 1 (DOCX 637 kb)

## Abbreviations

1D: One-dimensional domain
2D: Two-dimensional domain
MEP: Molecular electrostatic potential
SAS: Solvent-accessible surface
AR: Androgen Receptor
ARLBD: Androgen Receptor Ligand Binding Domain
PCa: Prostate Cancer
VS: Virtual Screening
EnsD: Ensemble Docking of Multiple Protein Conformations
IFD: Induced-Fit Docking
ROC: Receiver Operating Characteristic
