# Supplementary material for: Fragment-based Shape Signatures: a new tool for virtual screening and drug discovery
Source: J Comput Aided Mol Des. 2013 Dec 24;27(12):1009–36. doi: 10.1007/s10822-013-9698-7 (PMC3880490; doi:10.1007/s10822-013-9698-7)
Supplement: Supplementary file 1 — Supplementary material 1 (DOCX 637 kb) [file 10822_2013_9698_MOESM1_ESM.docx]

**Supplementary Electronic Information associated to:**

Title: "Fragment-Based Shape Signatures: A New Tool for Virtual Screening and Drug Discovery"

Journal: Journal of Computer-Aided Molecular Design - © Springer

Authors: Randy, J. Zauhar^*,§,^ Eleonora Gianti^§^ & William, J. Welsh^†^

^§^ Department of Chemistry and Biochemistry, University of the Sciences, 600 S. 43rd Street, Philadelphia, Pennsylvania 19104, United States

^†^ Department of Pharmacology, Robert Wood Johnson Medical School, University of Medicine and Dentistry of New Jersey, 675 Hoes Lane, Piscataway, New Jersey 08854, United States

Corresponding Author*: Dr. Randy J. Zauhar

Assoc. Prof. of Biochemistry and Director, Graduate Program in Bioinformatics

Department of Chemistry and Biochemistry

University of the Sciences in Philadelphia - 600 S. 43rd Street, Philadelphia, PA 19104

Phone: (215) 596-8691; FAX: (215) 596-8543; E-mail: [r.zauhar@usciences.edu](mailto:r.zauhar@usciences.edu)

**SM-1.** Structures of all 33 Hits generated from the Novobiocin screen ranked in Table1.
